# Breast histopathological image analysis using image processing techniques for diagnostic purposes: A methodological review

**DOI:** 10.1007/s10916-021-01786-9

**Published:** 2021-12-03

**Authors:** R Rashmi, Keerthana Prasad, Chethana Babu K Udupa

**Affiliations:** 1 Manipal School of Information Sciences, Manipal Academy of Higher Education, Manipal, India; 2 Kasturbha Medical College, Manipal Academy of Higher Education, Manipal, India

**Keywords:** Breast cancer, Histopathological images, Deep learning, Machine learning, H&E Stains, Image segmentation, Image classification

## Abstract

Breast cancer in women is the second most common cancer worldwide. Early detection of breast cancer can reduce the risk of human life. Non-invasive techniques such as mammograms and ultrasound imaging are popularly used to detect the tumour. However, histopathological analysis is necessary to determine the malignancy of the tumour as it analyses the image at the cellular level. Manual analysis of these slides is time consuming, tedious, subjective and are susceptible to human errors. Also, at times the interpretation of these images are inconsistent between laboratories. Hence, a Computer-Aided Diagnostic system that can act as a decision support system is need of the hour. Moreover, recent developments in computational power and memory capacity led to the application of computer tools and medical image processing techniques to process and analyze breast cancer histopathological images. This review paper summarizes various traditional and deep learning based methods developed to analyze breast cancer histopathological images. Initially, the characteristics of breast cancer histopathological images are discussed. A detailed discussion on the various potential regions of interest is presented which is crucial for the development of Computer-Aided Diagnostic systems. We summarize the recent trends and choices made during the selection of medical image processing techniques. Finally, a detailed discussion on the various challenges involved in the analysis of BCHI is presented along with the future scope.

## Introduction

**Fig. 1:**
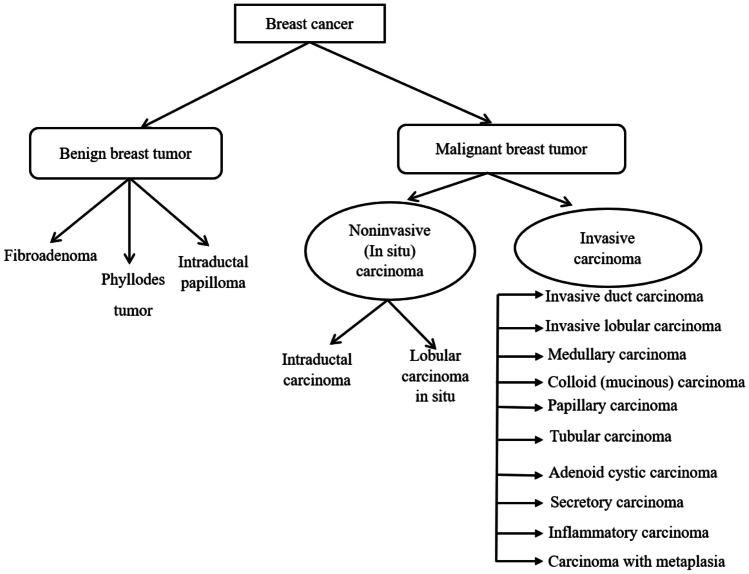
Histopathological types of breast cancer [10]

Cancer is a significant medical issue worldwide and is a major public health concern [1]. Among all the types of cancers, breast cancer in women around the world is the second most common cancer [2–4]. Breast cancer is a malignant lesion formation in the breast region. Early detection of breast cancer helps in better selection of treatment and prevents risk on human life. However, biopsy followed by histopathological analysis is the only way to determine with assurance the tumor is benign or malignant since it reveals the microscopic structure of the tissues. A histopathological analysis is a procedural work carried out in pathology laboratories to study manifestation of diseases in the tissues. Fine needle aspiration cytology is an alternative approach to histopathological image analysis that studies the structure and characteristics of cells. Fine needle aspiration cytology reveals the presence of tumour. However, the type of tumour cannot be decided by this analysis as it is not highly sensitive. Histopathological imaging has been considered as the gold standard in recognizing almost all sorts of cancers since it captures a more detailed view of the diseases [5, 6]. For accurate identification of breast cancer, biopsy accompanied by microscopic examination is an essential aspect. In a biopsy, a small section of tissue from the suspicious region of the body is removed, processed, and dyed with Hematoxylin and Eosin (H&E) stains. Hematoxylin stains the nuclei to dark purple or blue and Eosin stains other structures into shades of pink, red, and orange [7]. Subsequently, a pathologist studies the structure of the tissue from the stained glass slides to differentiate them as benign or malignant. Histopathological images of the breast are analyzed at different magnifications to study the cellular and tissue level variations [8]. For example, at 100x magnification, the tissue patterns and distributions are studied while at 400x magnification cytological features such as shape and size of the nuclei, hyperchromatic nuclei, mitotic cell, and prominent nuclei [9] are studied. Based on these features, pathologists classify the tumor slides as benign and malignant. In case of malignancy further examination is performed to grade the tumor and suitable treatment is given to the affected individual. Breast cancer can be of different types and each of these types has different microscopic features. Figure 1 shows the histopathological types of breast cancer.

**Fig. 2:**
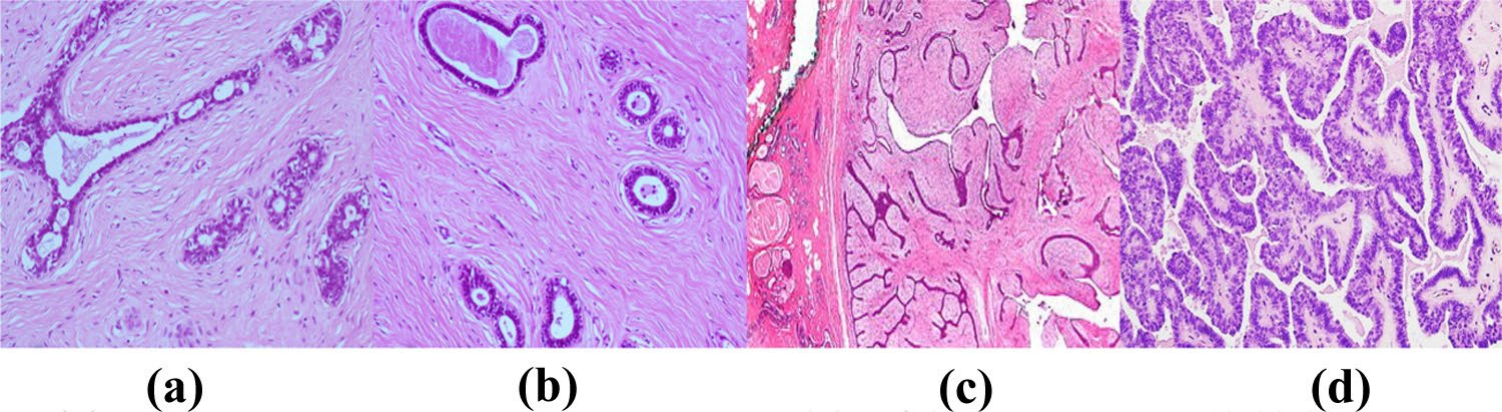
Microscopic patterns of benign breast tumor **(a)** Fibroadenoma (Intracanalicular pattern), **(b)** Fibroadenoma (Pericanalicular pattern) **(c)** Phyllodes tumor **(d)** Intraductal papilloma

**Fig. 3:**
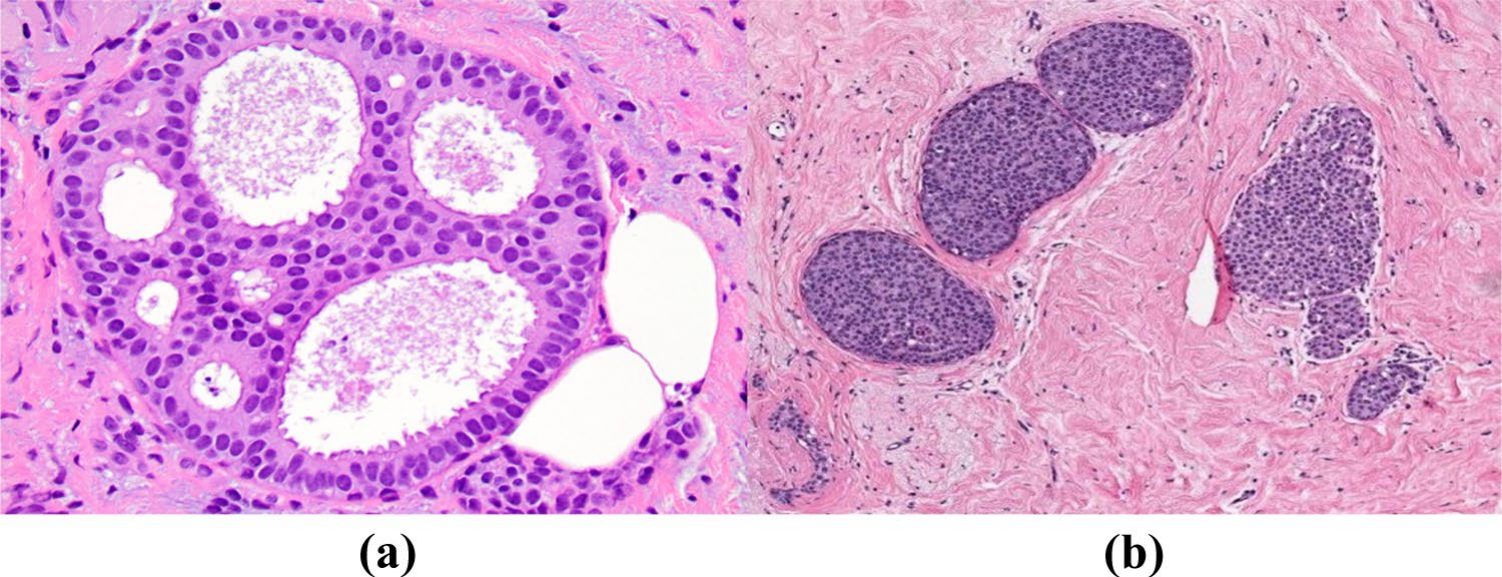
Microscopic patterns of Noninvasive (In situ) carcinoma **(a)** Intraductal carcinoma **(b)** Lobular carcinoma

**Fig. 4:**
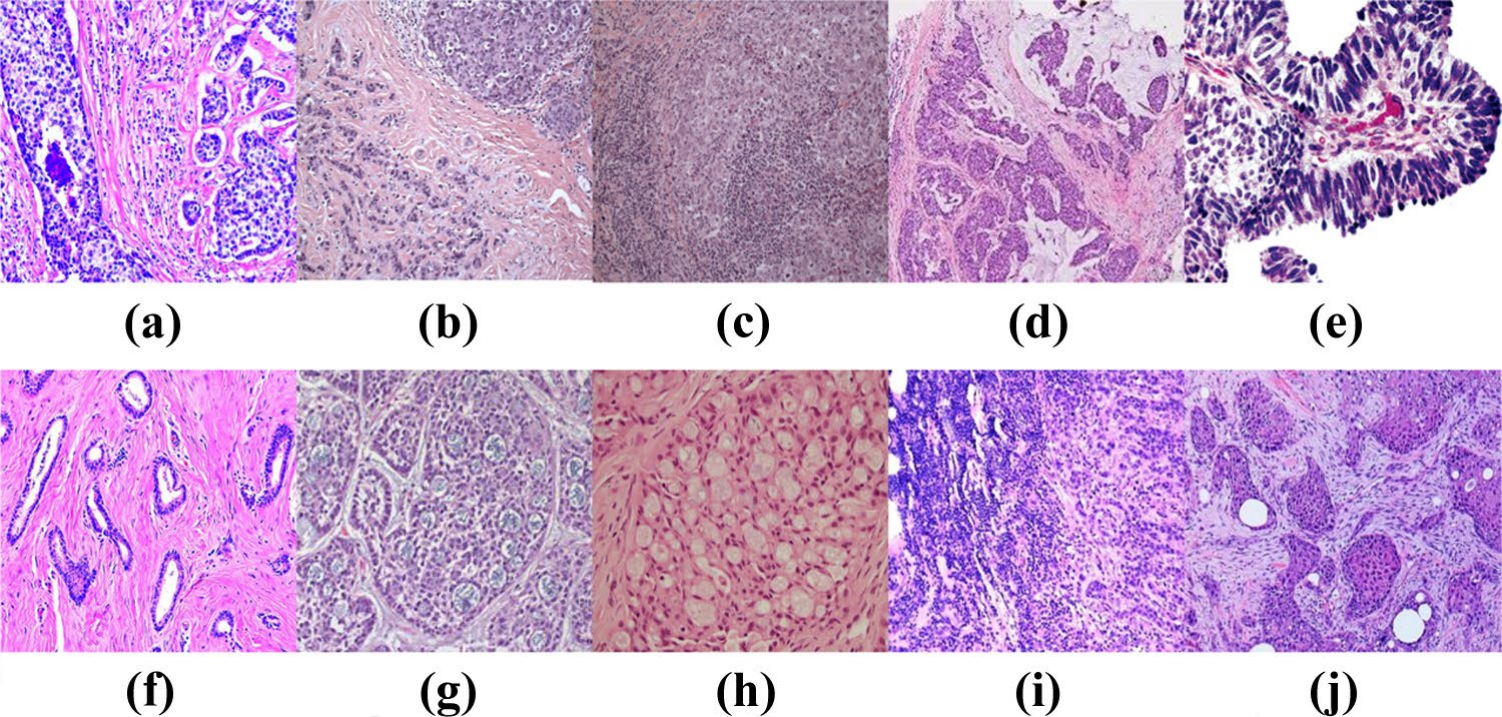
Microscopic patterns of Invasive carcinoma. **(a)** IDC **(b)** Invasive lobular carcinoma **(c)** Medullary carcinoma **(d)** Mucinous carcinoma **(e)** Papillary carcinoma **(f)** Tubular carcinoma **(g)** Adenoid cystic carcinoma **(h)** Secretory carcinoma **(i)** Inflammatory carcinoma **(j)** Carcinoma with metaplasia

During the manual evaluation, a pathologist examines the morphological features like shape, size, and color of the Region Of Interest (ROI) such as nuclei. Any change from the expected normal appearance of the nuclei is considered abnormal and further evaluation is carried out to confirm it as a malignant condition. In some cases, the pathologist also needs to report the tumor grading to know the aggressiveness of cancer [11, 12]. Figure 2 shows the microscopic patterns of benign breast tumors. Figures 3 and 4 represents the microscopic patterns of malignant breast tumor.

**Fig. 5:**
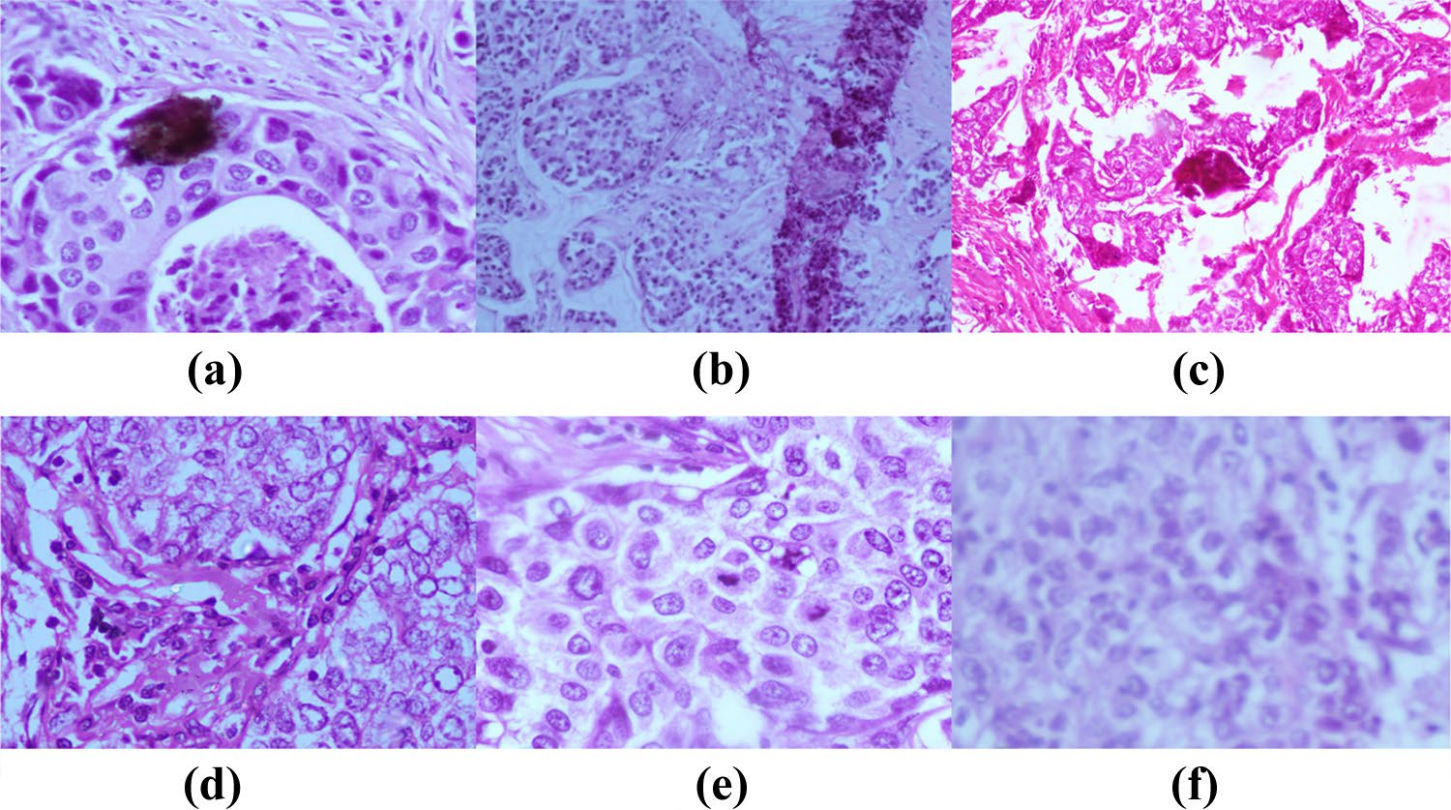
Histopathological image challenges. Figure **(a)** shows an example of artefact, **(b)** shows an example of tissue folding, **(c)** shows an example of thick sectioning, **(d)** shows an example of air bubbles, **(e)** shows an example of thin sectioning and **(f)** shows an example of blurring

From the past few decades, pathology laboratories are moving from optical microscopy to fully digital microscopy [13–15]. The evaluation is subjective and it may vary among pathologists and also among laboratories often leading to variability. The evaluation is also dependent on the experience and skill of the pathologist, instruments, staining procedure, and the approaches used to analyze the histopathological images. Manual evaluation of histopathological slides is a tedious and highly time-consuming task [7, 12]. Hence, there is a need for the design of an automation system to match the human evaluation process to diagnose abnormal cases correctly and act as a decision support system. Various image processing techniques are used to analyze images for evaluating the disease and prognosis. The basic steps involved in the development of Computer Aided Diagnostic (CAD) systems for histopathological images are pre-processing (color normalization), image segmentation, feature extraction, and classification [7, 8]. However, analyzing histopathological images is a challenging task in medical image processing due to the complex appearance, inconsistent staining, variation in illumination, overlapping and clustered nuclei and poorly fixed tissue samples [16, 17]. In the tissue preparation procedure, staining can also be affected by various determinants including the tissue itself, the thickness of the tissue section, the length of time at which tissue is exposed to stains, tissue foldings, artifacts in the stains [13], air bubbles [18] and blurring as shown in Fig. 5. All these factors result in poor segmentation and classification in the development of CAD systems. Over the past decades, much research is focused on the analysis of BCHI to bring automation to classify the image as benign and malignant using image processing and Machine Learning (ML) techniques [19–21]. This paper aims to provide a review of different attempts for automation of diagnosis based on histopathological image analysis using image processing techniques. In literature, several works [11, 12, 16, 21–23] reviewed breast histopathological image analysis. However, to the best of our knowledge, they focus only on a particular aspect of image analysis techniques such as color normalization [22, 23], segmentation [16] or classification [21]. In contrast to these works, the present paper focuses on all aspects of BCHI analysis such as datasets, color normalization, detection and segmentation of the potential ROI, feature extraction, and classification of histopathological breast images. This paper also reports the research gaps and concludes with an opinion on future work. The paper is restricted only to the review of the BCHI. The contributions of this review paper are as follows:

- The state-of-the review articles address a specific aspect of the problem such as segmentation of potential regions of interest or classification. However, we have summarized all the steps of BCHI analysis such as pre-processing, segmentation, feature extraction, and classification. Further, we have also summarized traditional and Deep Learning (DL) based methods to process BCHI.
- Developing DL models dependent on the availability of large datasets with annotations. In this regard, we have summarized various publicly available datasets.
- A summary on recent trends and popular choices of methods for various steps in processing BCHIs is provided.
- Finally, we have summarized, our observations, all the challenges of processing images along with the future direction.

This paper is structured as follows; “Publicly available datasets” gives details about publicly available datasets for BCHI analysis. “Overview of the review articles on automation of histopathological image analysis” summarizes the review that was carried out in the field of histopathological image analysis. The review of various image analysis approaches used for automation is discussed in “Image processing approaches”. “Discussion” and “Challenges” briefs about the discussion and the future work. Finally, the paper ends with a conclusion.

## Publicly available datasets

To develop a robust CAD system for breast cancer detection using histopathological images, it is necessary to have image datasets. The details of the publicly available datasets for BCHI analysis are given in Table 1. Details such as the total number of images, magnification factors, image size, image format, and classes are highlighted. These image datasets can be used by the researchers to develop an algorithm for the classification of Breast Cancer Histopathological Images (BCHI). However, all these datasets provide annotations at the image level for image classification. This limits the application of image processing methods and analysis of histopathological images at the pixel-level.

BreakHis [24] database is the most popularly used database among the research community for classification. BreakHis database contains a total of 7909 images collected from 82 patients organized into four different magnification factors namely 40X, 100X, 200X and 400X. A total of 2480 images of benign and 5429 images of malignant cases of various subtypes of BCHI are provided. The histopathological types of benign breast tumors are adenosis, fibroadenoma, phyllodes tumor, and tubular adenoma. The types of malignant breast tumors are ductal carcinoma, lobular carcinoma, mucinous carcinoma, and papillary carcinoma. This dataset allows the researchers to address the problems in terms of binary and multi-class classification tasks and also at different magnifications.

The second popularly used database among the research community is BreAst Cancer Histology (BACH) dataset [25]. The dataset is composed of microscopy images and Whole Slide Images (WSIs). A total of 400 microscopy images and 30 WSIs of multiple regions namely normal, benign, in situ carcinoma, and invasive carcinoma are provided. MITOS-ATYPIA-14 [26] is the most frequently used database for the detection and classification of mitosis and non-mitosis [27]. BACH [25], TUPAC-2016 [27], Camelyon 16 [28] and Bioimaging 2015 [29] databases are also used by the researchers for the analysis of BCHI.

**Table 1:** Overview of the publicly available BCHI datasets

**Database Name Ref**	**Total no. of images**	**Magnification**	**Image details (image size and format)**
BreakHis [24]	7909	40X, 100X, 200X, 400X	Benign=2480, Malignant=5429
			700*460 pixels
			PNG format
IDC [30]	162	40X	198,73=IDC negative,
			78=IDC positive patches from 162 slides
			1360*1024 pixels
			tiff format
BACH [25]	430	-	400=Microscopy images (2048*1536 pixels)-image-wise label
			30= Whole-slide images (42113*62625 pixels)- pixel-wise label
			tiff format- microscopy images
			in.svs format- WSI
TUPAC-2016 [27]	821	40X	500=training
			321=testing
Camelyon- 2016 [28]	400	40X, 10X, 1X	WSIs of sentinel lymph node of breast cancer
Camelyon- 2017 [31]	200	40X	WSIs of sentinel lymph node of breast cancer
MITOS-ATYPIA-14 [26]	-	20X,40X	284 frames at 20X magnification,
			1136 frames at 40X magnifications
			tiff format
Bioimaging 2015 [29]	-	200X	249=training, 20=testing and 16 extended test datasets
			2048*1536 pixels
BreCaHAD [32]	162	-	1360*1024 pixels tiff format
Breast cancer	151	-	WSI images of breast cancer semantic segmentation [33]
NuCLS [34]	151	-	WSI images of breast cancer

## Overview of the review articles on automation of histopathological image analysis

From the literature, it is observed that a few researchers have carried out a review on histopathological image analysis [11, 12, 35], stain normalization [22, 23], segmentation [16] and classification [21]. The details about the review on the histopathological image analysis are discussed and summarized in this section.

Gurcan et al. [11] summarized the recent state-of-the-art CAD technology for histopathological image analysis. The authors have also emphasized the usage of standard datasets for the evaluation of developed CAD systems since it helps in easier analysis and comparison. Veta et al. [12] reviewed various methods proposed for the analysis of BCHI. The authors discussed the complexity of the tissue characteristics that need to be studied to improve the robustness of the system. The authors in [17, 19–21] reviewed the usage of ML techniques for the analysis of histopathological images. In [20] and [21], a summary of the available dataset for breast cancer analysis and generalized image classification techniques like supervised, unsupervised, and DL classifiers is provided. The different approaches used for the histopathological image analysis like nuclei detection, segmentation, feature extraction, and classification were reviewed by Irshad et al. [16]. They also discussed a few benchmark datasets, the problems and challenges of microscopic image segmentation, and mentioned the issue of robustness in terms of clinical and technical conditions. He et al. [7] discussed the characteristics of the histology images and reviewed the state-of-the-art methods used for image segmentation techniques for feature extraction and disease classification. In [8] the authors reviewed the computational steps required to automatically diagnose cancer in histopathological images. In their review, they investigated the types of features that are used in the diagnosis of different types of cancer. Janowczyk et al. [36] investigated how DL approaches can be used in the digital pathology domain. The study was conducted on the set of use cases for segmentation, detection, and classification. It has been suggested that the quality of the classifiers can be improved by utilizing hand-crafted features along with the DL approach. Various image analysis methodologies in histology image analysis were surveyed by Loukas et al. [37]. The authors described the cell detection problem and also listed out the limitations that need to be addressed. Fuchs et al. [38] reported the challenges involved in computational pathology workflow. They discussed the future directions in research for diagnostic ML. State-of-the-art methods and applications involved in large-scale medical image analytics were summarized by Zhang et al. [39]. Litjens et al. [40] surveyed the DL techniques in the domain of medical image analysis. The authors discussed the state-of-the-art DL approaches and challenges involved in the analysis of BCHI. A comprehensive survey on the automatic diagnosis of breast cancer using DL techniques on the BreakHis dataset was provided by Benhammou et al. [41]. They also explored the DL technique for magnification-independent multi-category classification problems. The authors in [42] presented a review on lymph node assistant for breast cancer images. The findings from a multi-reader and multi-case study of pathologists utilizing state-of-art algorithms are summarized. Debelle et al. [43] reviewed state-of-the-art DL algorithms used for the detection of breast cancer. Srinidhi et al. [44] presented a comprehensive overview of deep neural network architectures developed for analyzing histopathological images and also outlined some of the issues and future trends. The ML and DL approach for diagnosis of breast carcinoma were surveyed in [45] where they discussed the issues involved in the development of CAD systems. Further, they analyzed various ML and DL approaches for cancer diagnosis.

From the literature, it is seen that a considerable amount of review is carried out on the various aspects of automation of histopathological images. However, there is a lack of detailed review focusing on all aspects of histopathological image analysis such as color normalization, detection, and segmentation of the potential ROI, feature extraction, and classification in literature. To the best of our knowledge, these review papers are specific and focused on a particular aspect of breast histopathological image analysis.

## Image processing approaches

Histopathological image analysis aims to classify the images as malignant and benign and act as a decision support system. Analysis of histopathological images by using image processing techniques involves various steps such as color normalization, segmentation, feature extraction, and classification. This pipeline of operation is popularly used in traditional image processing techniques while modern approaches based on DL use end-to-end style learning. In the traditional approach, color normalization step is used as a pre-processing step to remove the variations in color and illumination. Subsequently, a segmentation algorithm is applied to identify potential regions of interest. Feature descriptors are defined to extract the most discriminative features and finally, an ML algorithm is trained to classify the images into different classes based on the extracted features. 

This section initially presents various color normalization techniques developed followed by detection and segmentation of the potential regions of interest. Subsequently, a summary of various feature extraction and classification techniques used for BCHI is presented.

### Color normalization

**Fig. 6:**
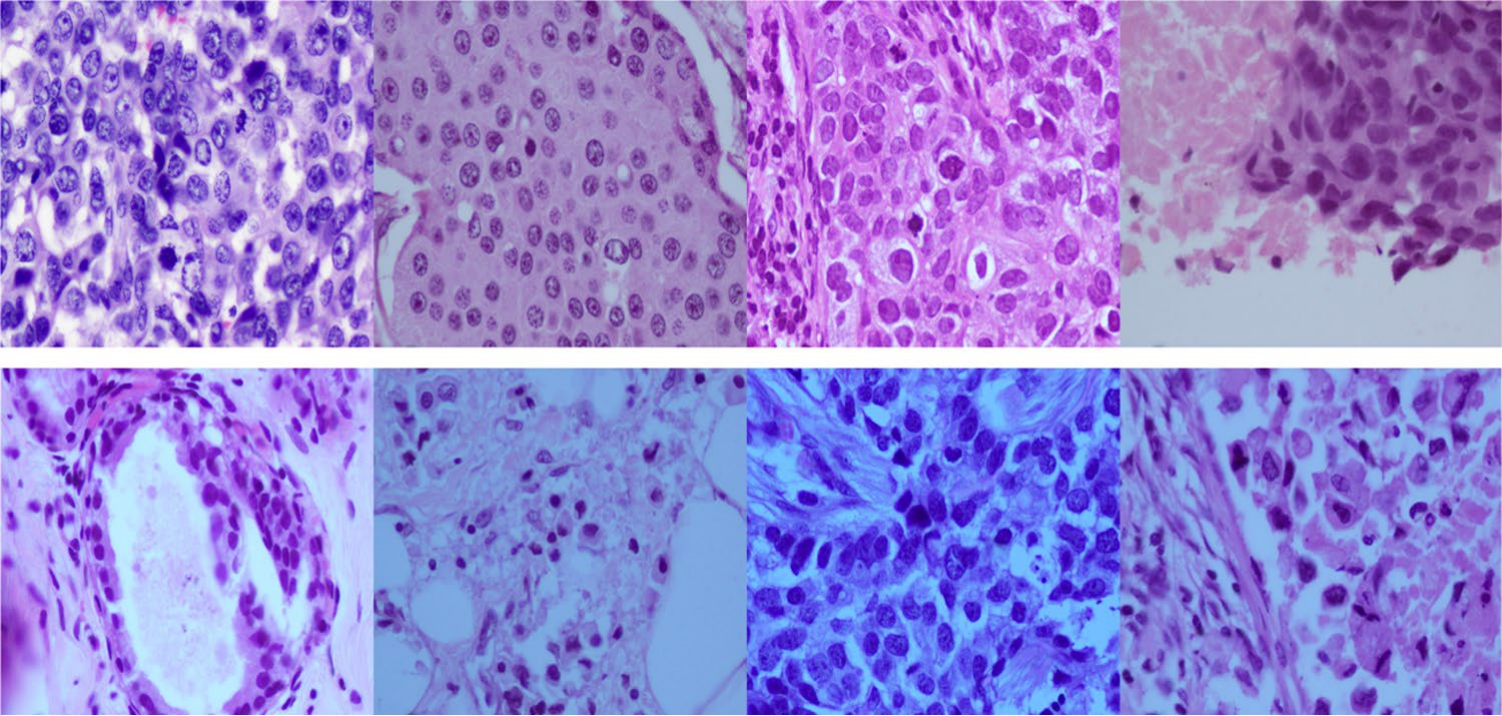
Sample images to demonstrate the colour shade and illumination variations

Due to inconsistency in staining and image acquisition, histopathological images suffer from color and illumination variations. The main factor that influences the color variation is the difference in staining procedures adopted in different laboratories [22, 23, 46]. The different scanners and types of equipment used to capture the image results in illumination variation. Ignoring the variance in color and illumination of histopathological images often leads to incorrect results [47]. To overcome these problems pre-processing step, known as color normalization is employed for the histopathological image analysis. An illustration of color and illumination variance of BCHI are shown in Fig. 6. In this section, a summary of various works on the color normalization techniques for histopathological images is presented.

Many attempts have been made to eliminate the color and illumination variations in histopathological images over the past years [48–51] , [52–54]. Systematic study on assigning of an image color to another image was carried out in 2001 by Reinhard et al. [55]. The color characteristics of a template image are brought to an input image based on mean and standard deviation in LAB color space representation. This method is popularly known as the Rehinard method. To improve the color consistency in histopathological images Basavanhally et al. [48] proposed an Expectation-Maximization (EM) based segmentation–driven Standardization (EMS) algorithm. In this approach, the input and the normalized image were qualitatively compared during the nuclei segmentation step. To obtain an accurate stain density map in histopathology images Vahadane et al. [51] proposed a Structure-Preserving Color Normalization (SPCN) algorithm. They converted the source and the target RGB images to optical density space using the Beer-Lambert law. The structure of the original image is preserved by employing the SPCN algorithm. But this method fails to preserve the color information of the source image and suffers from local minima. Roy et al. [50] developed a Fuzzy based Modified Reinhard (FMR) method to handle the color variations in H&E stained images. The conventional Reinhard method reduces the contrast of the source image. Hence to overcome this problem, fuzzy logic with the Reinhard method was incorporated. The authors of [49] experimented on different color normalization algorithms and proposed stain normalization using saturation-weighted statistics. Their method is capable of identifying the cause for color variation. An alternative reference space for color normalization in histopathological images was reported by [54]. Euclidean distance was utilized to measure the intra-cluster and inter-cluster ratios. Each eight-bit input image is reduced to ten colors using K-means clustering. To overcome inconsistency in the staining process, Macenko et al. [52] proposed a stain quantization algorithm that belongs to a class of unsupervised normalization approaches. The images were analyzed on the shape and stain-based features. Further, an algorithm to obtain the optical stain vector with various stain combinations was provided. Tosta et al. [53] developed an estimation method to handle the faded regions in the histopathological images. The downside of the proposed method is that fails to preserve the tissue color corrections resulting in low performance. A Quantile normalization approach was proposed by Cao et al. [56] to develop an automatic breast cancer grading system. Gadermayr et al. [57] investigated the various stain normalization techniques concerning tissue classification. The study included five different normalization techniques, followed by extraction of five features on two differently stained renal images on two experimental setups. Khan et al. [58] proposed a non-linear mapping technique for the normalization of an input image to the color distribution of the reference image based on color deconvolution. Bukenya [59] developed a hybrid technique for stain normalization which consists of two stages namely, the stain separation stage and color transfer stage. The input images and the reference image were converted from RGB to Optical Density space and performed stain separation and color transfer steps to normalize the image. A stain deconvolution approach by employing a multi-resolution wavelet representation of the image to evaluate the stain mixing matrix was presented by Alsubaie et al. [60]. They converted the input image to optical density image and extracted R, G, and B channels, and decomposed each color channel to its sub-band by employing wavelet decomposition. In the last few years, much study is carried out to address the problem of stain normalization in terms of processing time and additional system memory utilization. Anghel et al. [61] proposed an unsupervised approach for stain normalization in WSI. They adopted the method proposed in [52] to perform a high-performance stain normalization system and also proposed a method to detect low-quality images. The main objective of the study was on optimizing and enhancing the robustness of the stain normalization algorithm. An automated color segmentation approach was developed by Kothari et al. [62]. The experiment was performed on four different types of H&E stained images. The images were normalized by adopting two types of normalization techniques namely quantile normalization on all the pixels and normalization on the color maps of the images which are obtained by extracting the unique color. A color segmentation accuracy of 85% was reported. An experiment on extracting the color and texture information to evaluate the need for stain normalization was conducted by Gupta et al. [63]. The authors suggested a method for the selection of reference images. A comparative study on different normalization techniques for epithelium and stromal classification was performed by Sethi et al. [64]. A multi-resolution segmentation approach was performed for super pixel-based classification. The patch-based classification was performed by using Convolutional Neural Network (CNN). Bejnordi et al. [65] developed a fully automated algorithm called whole-slide image color standardizer for the standardization of whole-slide histopathological images. Color and spatial information were employed to classify the image pixel into distinctive stain components. In [66], a pipeline that utilizes an unsupervised method based on stain vector estimation was proposed to handle the memory and runtime bottlenecks in high magnification images. They claimed the method is computationally less expensive in terms of memory. Magliaro et al. [67] developed an open-source tool called Histology for the separation of dye colors in histology images. The graphical user interface was developed by utilizing the K-means clustering algorithm to isolate the dye colors in histological images. The performance of the tool was compared with the ImageJ color deconvolution plugin in terms of speed of color separation. An algorithm to reduce the stain variations in H&E stained histopathological images was proposed by Bejnordi et al. [46]. The standardization algorithm is based on the clustering of the images into two tissue components. Bautista et al. [68] implemented a color correction method by utilizing the color information of nine color patches of a color calibration slide. The proposed method does not work when there are color variations caused by the staining procedure. A flexible and robust image analysis algorithm was developed for the separation and quantification of immunohistochemical staining by Ruifrok et al. [69]. The proposed algorithm was designed to deconvolve the color information captured with RGB cameras and to estimate the contribution of each of the applied stains. A color transfer approach based on YCbCr color space for the enhancement of peripheral blood smear images was developed by Prasad et al. [70]. An approach for the selection of the template image and the effect of the good template image on color normalization was described. Clarke et al. [71] developed a color calibration assessment slide for digital pathology. The positive aspect of the method is the variations in the tissue thickness does not alter the shape of the spectrum. The authors also claimed that the proposed method will add to the reproducibility of the automation of image analysis systems. Bautista et al. [72] proposed a method for the detection and visualization of tissue folds in pre-scanned WSI. The proposed method incorporates the color enhancement technique which distinguishes between the folds and non-fold regions in an image. Color saturation and luminance of the image pixels with weighted differences are used as a shifting factor to the input RGB color of the image. In recent years color normalization was also carried out by using DL techniques [73, 74]. Generative Adversarial Networks (GANs) model for color normalization of H&E stained images was developed by Zanjani et al. [74]. The model has an end-to-end framework that was trained to learn the chromatin space of H&E images. Janowczyk et al. [73] proposed a stain normalization technique using Sparse Autoencoders (StaNoSA) to handle the color variations in H&E stained images. The StaNoSA was developed by incorporating sparse encoders as a core method to segregate the input images into distinct tissue classes. Hamidinekoo and Zwiggelaar [75] proposed a DL-based network to detect mitosis in BCHI. Color normalization was performed by utilizing the RGB histogram specification method. In this method, color values of the target image are normalized to the source image on a pixel-by-pixel basis.

### Segmentation

Segmentation methods divide an image into a smaller group of pixels. Application of segmentation algorithms to BCHI aids in identifying the ROI . In this section, we provide details of the state-of-the-art methods for ROI detection and segmentation.

#### ROI detection and segmentation using a traditional approach

**Fig. 7:**
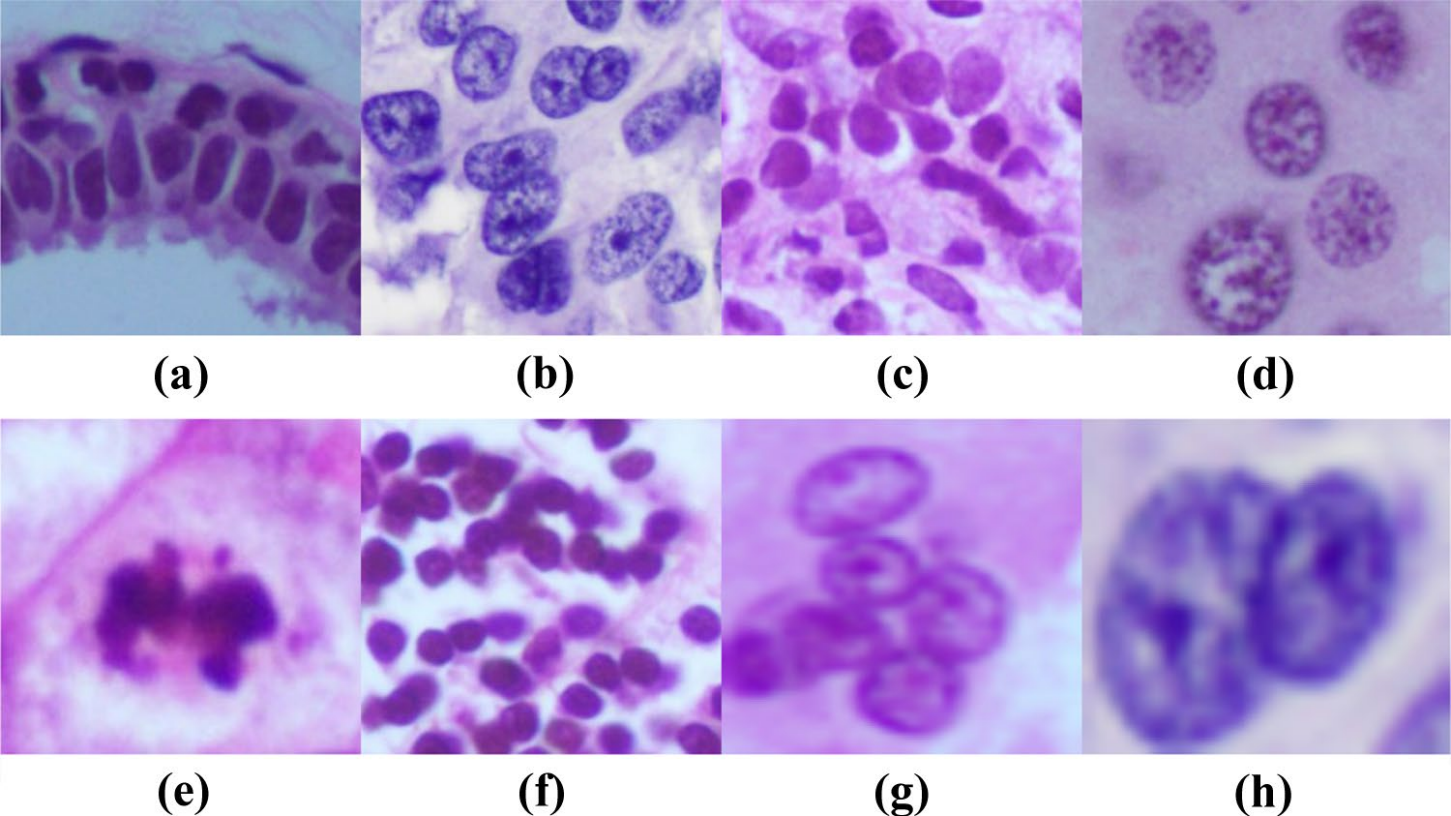
An example of **(a)** normal nuclei, **(b)** prominent nucleoli, **(c)** hyperchromatic nuclei **(d)** cancerous nuclei, **(e)** mitotic nuclei, **(f)** lymphocyte, **(g)** clustered nuclei and **(h)** overlapping nuclei

Segmentation of the ROI from histopathological images has received much attention in the past decade. Many approaches have been proposed in the literature for ROI detection and segmentation. Traditional image processing methods utilize thresholding [76], watershed transform [77–79], active contour models [80] and ML for nuclei segmentation. Figure 7 shows various types of nuclei in the BCHI. The state-of-the-art methods for ROI segmentation and detection using traditional approaches are summarized in this section.

Veta et al. [79] presented a marker-controlled watershed-based technique to segment the cancerous nuclei in whole digital slide images of the breast. A positive predictive value of 0.90, a sensitivity of 0.83, and dice coefficients of 0.9 were reported. Fatakdawala et al. [80] considered HER2+ breast histopathological images to detect the lymphocytes. They proposed expectation-maximization-driven geodesic active contour with overlap resolution. The method was evaluated on a total of 100 images were reported a detection sensitivity of over 86% and a positive predictive value of 64% respectively. Paramanandam et al. [81] proposed an integrated framework by utilizing a gradient-driven voting mechanism using 2D tensor voting along with the Markov Random Field loop backpropagation technique to segment the single nuclei breast images. A total of 8 H&E stained images along with two WSI were used in their study.

Wang et al. [82] developed an automatic nuclei segmentation method based on multi-scale region-growing with a double-strategy splitting model. To enhance the contrast between the nuclei and background, the top-hat transform method was used. Adaptive mathematical morphology and curvature scale space methods were used to separate the overlapped nuclei. A segmentation accuracy of 91% was reported. Dundar et al. [77] developed a method to classify intraductal breast lesions from histopathological images. The input images were converted from RGB to LAB space and segmentation was performed by using a watershed-based segmentation algorithm to segment the single cells. A total of 149 ROIs were used for testing and were reported an overall accuracy of 87%. Four different clustering algorithms are compared and studied for the task of nuclei segmentation by Kowal et al. [76] from fine needle biopsy images of the breast. Adaptive thresholding is used to segment the foreground and background objects. A clustering algorithm was applied to identify the nuclei in the foreground objects. Further, 42 morphological, texture, and topological features were extracted from the segmented nuclei. Three different classifiers were used for classification. A classification accuracy ranging from 96-98% was reported. Kost et al. [78] proposed a method for automatic nuclei detection using probability maps and watershed-based segmentation algorithms. Hematoxylin channel is extracted from the input images to generate a probability map, which represents the nuclei for each pixel. A threshold of 0.5 was utilized to detect the nuclei. An extended watershed-based algorithm was used to reduce over-segmentation and reported F1-scores ranging between 0.83-0.93. Vink et al. [83] proposed a method for detection of the nucleus from breast tissue using AdaBoost and active contour. They focused on the detection of nuclei of the epithelial cells, lymphocytes, and fibroblasts. The detection accuracy of 95% was reported. An integrated method for gland and nucleus detection and segmentation was proposed by Naik et al. [84]. The segmentation was performed using image information at three scales. The architectural features were used to distinguish cancer and non-cancer breast histopathological images. A total of 18 benign and 36 malignant images were considered. An accuracy of 81% using automatic segmentation and 77% with manually segmented structures were reported. Petushi et al. [85] performed segmentation by using adaptive thresholding and morphological operations on the grayscale image. Graph-based features were utilized to distinguish the lymphocyte and cancer nuclei by Basavanhally et al. [86]. Lymphocyte was detected and segmented using a segmentation scheme consisting of a Bayesian classifier and template matching for a total of 41 images. An unsupervised learning approach to identify the ROIs in the malignant samples of WSIs was proposed by Kumar and Prateek [87]. K-means clustering, morphological features, and shape features were utilized to identify the ROI. An accuracy of 85% was reported.

Bejnordi et al. [88] proposed a multi-scale superpixel classification algorithm for the identification of epithelial areas in WSIs. The ROI was segmented from the detected epithelial regions using a graph-based clustering algorithm. Authors in [89] proposed a method to differentiate between neoplastic epithelium and stromal reaction in breast carcinomas. Density-based clustering was performed on the centroid of the tumor cell to segment the neoplastic epithelium. The proposed algorithm was evaluated on 100 H&E stained images. An F1 score of 0.88 and a mean Jaccard index of 0.84 was reported. Paul and Mukherjee [90] reported a method to segment mitotic cells by adopting relative-entropy maximized scale space with morphological operations. Paramanandam et al. [91] focussed on the detection and segmentation of tubule regions in the images using K-means clustering algorithm. Colour-based segmentation and grid analysis was utilized to identify the nuclei regions. Filipczuk et al. [92] reported an automatic diagnostic approach for the analysis of fine needle biopsy images. Circular hough transform and Support Vector Machine (SVM) was used for the detection of nuclei. The model was evaluated on 737 microscopic images and reported an accuracy of 98% respectively.

#### ROI detection and segmentation using DL approach

Recently, DL-based algorithms have gained much popularity in the medical imaging community due to their end-to-end style learning. DL methods are attracting widespread interest in nucleus detection from histopathological images as they can learn deep features [93]. But these DL algorithms are dependent on the availability of large datasets with annotations. Several authors have utilized DL-based methods to segment nuclei from breast histopathological images [94–99]. The state-of-the-art methods for ROI segmentation and detection using DL approaches are summarized in this section. Naylor et al. [96] used deep neural network architecture with mathematical morphology for segmentation of nuclei. The three well-known architectures namely Pang Net, Fully Convolutional Net, and Deconv Net were utilized for semantic segmentation. A total of 33 images with 2754 annotated cell nuclei were used for the study. Segmentation results ranging from 76-94% were reported. A deep CNN-based shape recognition was introduced by Xing et al. [98] to generate initial shapes, which learn hierarchical feature representation from raw images. The algorithm was tested on three different types of pathology datasets. The precision of 71%, recall of 88%, and F1-score of 78% were reported. A Stacked Sparse Autoencoder with an encoder and decoder network for efficient detection of nuclei for histopathological images was developed [99]. From the pixel intensities, the model learns high-level features to distinguish the features of nuclei. F-measure of 84% and average area under the Precision-Recall curve of 78% were reported. Metha et al. [95] implemented an encoder and decoder model to semantically segment the tissue labels in breast images. A total of 240 breast biopsy images were used. An overall segmentation accuracy of 93% was reported. 

Jung et al. [94] proposed the Mask R-CNN segmentation framework to achieve nuclei segmentation. Color normalization was performed by utilizing U-Net based deep convolution Gaussian mixture model. In the post-processing step, multiple inferences were utilized to improve segmentation performance. Wang et al. [97] developed a bending loss regularization architecture for nuclei segmentation. The network is composed of an encoder and a decoder model. The pre-trained 50 layer Residual Neural Network (ResNet) was used as an encoder. The nuclei instance branch and distance map branch were utilized in a decoder model. The study was performed on 21000 annotated nuclei from 30 images. 

A method to segment the touching and overlapping nuclei was studied in [100–105]. In [103] Deep Convolutional Neural Network (DCNN) and marker-controlled watershed techniques were combined to segment the overlapping nuclei. Deep Interval-Marker-Aware network architecture was designed for learning the foreground, marker, and interval of nuclei. The logical operations were utilized to get foreground results of the nuclei from the learned interval between the overlapping nuclei. The results of the learned marker and nuclei segmentation refined by interval are passed to the marker-controlled watershed for separating the overlapping nuclei. Kumar et al. [101] performed nucleus segmentation using CNN. The architecture was designed to produce a ternary map that was able to identify inside and outside nucleus along with the nuclear boundary containing those between the touching and overlapping nuclei. The authors in [100] designed a CNN to detect the Invasive Ductal Carcinoma (IDC) tissue regions in the WSIs. A total of 162 WSI slides were used to evaluate the method. They reported an F-measure and accuracy of 71% and 84% respectively. Xu et al. [104] presented a CNN for the detection of nuclei, a region-based active contour method for segmentation, and adaptive ellipse fitting to handle the clustered and overlapping nuclei. The DL architectures such as Residual-inception-channel attention U-net [105], Atrous spatial pyramid pooling U-net [102], and conditional GAN [106] were also explored for the nuclei segmentation.

Several studies have been carried out to detect and segment mitosis in BCHI [107–110]. Wahab et al. [110] proposed a transfer learning-based deep CNN for segmentation and detection of mitotic nuclei. Pre-trained CNN’s were used for segmentation. They reported the detection rate with an F1-measure of 0.73 and area under the precision-recall curve as 76% respectively. Das and Dutta [107] reported a method to detect mitosis in BCHIs using DCNN model. The Haar wavelet was utilized to decompose the input image patches of 81 * 81 pixels to patches of $21\times 21$ pixels. A total of 70 images were considered for training which has 720 mitotic cells and 30 images were considered for testing containing 200 mitotic cells. Precision, recall, and F1-score of 84%, 83%, and 85% were reported on the testing dataset. The authors in [109] proposed a Mask RCNN to automatically detect and segment mitosis in breast cancer slides. A multi-stage DL framework was reported by Li et al. [108] to detect the mitotic cells. The framework consists of a deep segmentation network, deep detection network, and deep verification network.

A study was also performed for automatic segmentation of carcinoma distribution in WSI of breast tissues [111]. Initially, WSI’s were split into patches and segmented by using DCNN along with encoder and decoder model. The merging technique based on fully connected Conditional Random Field was applied to combine the segmented patches. Segmentation accuracy and frequency weighted intersection over union (FWIoU) of 95% and 92% respectively were reported. The performance of a DL algorithm to detect the lymph node metastases in H&E stained breast cancer tissue sections was evaluated in [112].

**Fig. 8:**
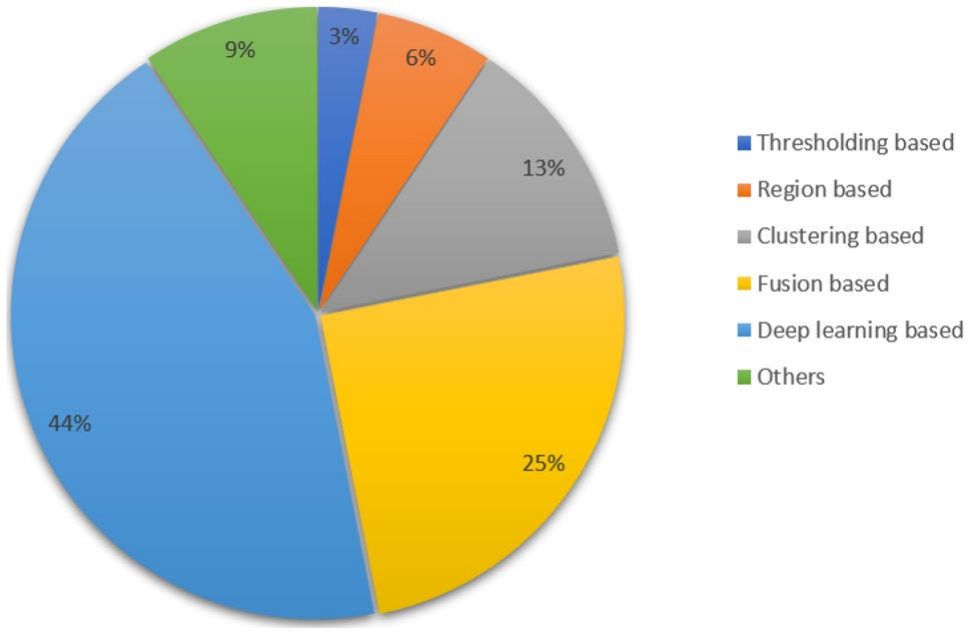
Illustration of segmentation methods used in literature

A summary of the different segmentation techniques used in the literature for ROI detection along with the achieved performance metrics is given in Table 2. Figure 8 represents the different segmentation methods used in the literature. We have categorized the segmentation methods into threshold-based, region-based, clustering-based, fusion-based, and DL-based techniques. In the “Others” category, we have included the methods such as level set information and color-based segmentation. It is observed from Fig. 8, that most of the research groups used DL-based techniques for detection and segmentation of the ROIs in BCHI.

**Table 2:** Summary of the approaches used for ROI segmentation in BCHI

Segmentation method (Generally categorization)	Segmentation method (Particular categorization)	**ROI**	**No of images**	**Evaluation Metrics**
Threshold-based method	Adaptive thresholding [76]	Cancer Nuclei	24 H&E images	NA
Region-based method	Marker-controlled watershed-based [79]	Nuclei	39 images	PP=90%Sen = 83%
				DC = 0.9
	Watershed-based [78]	Nuclei	26 cells	F1-score=0.93
**Clustering-based**	Graph-based clustering [88]	Epithelial areas in WSIs	75=benign	NA
	Density-based spatial clustering [89]	Neoplastic epithelium	75=DCIS	F1-score=0.88
	K-means clustering [87]	Nuclei	100 H&E images	Mean Jaccard
				index = 0.84
				Accuracy = 85%
	K-means clustering [91]	Tubule	10 H&E WSIs	Accuracy = 90%
			29 H&E images	
Fusion-based method	Gradient driven voting mechanism +	Nuclei	8 H&E WSI	Precision=93%
	Markov Random			Recall=96%
	Field loop backpropagation [81]			DC=0.9
	Wavelet decomposition + multi-scale region-growing [82]	Nuclei	32=Normal cell 22=Cancer cell	Accuracy=91%
	Expectation–maximization (EM) driven geodesic active contour+ overlap resolution [80]	Lymphocytes	100 images	Sen=86%
	Clustering +watershed-based [77]	Nuclei	149 cells	Accuracy =87%
	AdaBoost+active counter [83]	Nuclei	NA	Accuracy=95%
	Adaptive thresholding + Clustering [76]	Nuclei	24 H&E images	NA
DL based	DNN=Pang Net, Fully Convolutional Net, Decon Net [96]	Nuclei	2754 annotated nuclei	Accuracy =95% Recall=90% IU =81% Precision=86% F1-score =80%
	Stacked Sparse Autoencoder [99]	Nuclei	3500 nuclei from 500 images	F1-score=84%, Precision-Recall Curve=78%
	Encoder and decoder model [95]	Tissue labels	240 biopsy images	Accuracy=93%
	Mask R-CNN [94]	Nuclei	33 images of 512X512	Precision=91% F1-score=0.86
	Bending loss regularization network [97]	Nuclei	21000 nuclei (4 breasts)	DC = 0.81
	DCNN +Encoder and decoder [111]	Tissues	12 breast cancer WSI	FWIoU= 95%
	CNN [100]	IDC	162 WSI slides	F-score =71% Accuracy= 84%
DL based	CNN+ Active counter+ Adaptive ellipse fitting [104]	Nuclei	204WSIs	F1-score=80-85% AveP=74-82%
	Residual-inception-channel attention U-net [105]	Nuclei	TCGA dataset	F1-Score=0.82
	Atrous spatial pyramid pooling U-net [102]	Nuclei	NA	NA
	Conditional Generative adversarial network [106]	Nuclei	NA	F1-Score=0.86
	Transfer learning based-deep CNN [110]	Mitosis cell	NA	F1-Score=73% Precision_recall=76%
	DCNN [107]	Mitosis cell	920 mitosis cells	Precision=0.84% Recall=0.83 F1-score=85.05
Others	Level set information [84]	Nuclei	18=Benign 36=Malignant	Accuracy=81%
	Hybrid level set information [56]	Nuclei	4000 Nuclei	NA
	Color-based [62]	NA	TCGD dataset	Accuracy=85%

NA= Not available; PP=Positive Predictive; Sen= Sensitivity; DC=Dice Coefficient; FWIoU= Frequency Weighted Intersection over Union; AveP= Area under Precision recall curve

### Classification

Feature is defined as an “interesting” part of an image and helps in describing different regions in an image. These features help in identifying the same objects in different images. Classification is a process of assigning labels to different groups based on the identified features. In this section, we provide details of the state-of-the-art methods used for feature extraction and classification.

#### Classification using traditional image processing approach

In the last few years, many studies were carried out to extract different features from BCHI. The handcrafted features such as statistical features, different texture features, morphological features, and color features were studied to classify the images as benign and malignant.

An automatic method to classify stromal regions according to their maturity was studied by Reis et al. [113]. At Multiple scales, basic image features and Local Binary Patterns (LBP) were extracted. Random decision tree classifier was used to classify the stromal regions. A total of 55 H&E stained images of invasive breast carcinoma were used in the study. Classification accuracy of 84% was reported. A content-based histopathological image retrieval framework was proposed by Zheng et al. [114]. The classification and retrieval accuracy of 94% was reported. A combination of features to classify breast cancer tissues was studied in [115]. Curvelet and LBP features were extracted from the images. SVM, random forest, decision tree, and polynomial classifier were used for classification. A method to automatically detect and grade the lymphocytes in HER2+ BCHI was proposed by Basavanhally et al. [116]. Region growing and Markov random field algorithms were utilized for the detection and SVM used for classification. The proposed method was evaluated on a total of 41 images. Classification accuracy of 90% was reported.

The authors in [117] addressed the problem of grading invasive breast carcinoma using Grassmann manifold. A vector of locally aggregated descriptors encoding technique was designed. The classification accuracy of 95% was reported on their dataset and 91% on BreakHis dataset. Das et al. [118] proposed a dictionary-based approach for nuclear atypia scoring. The methods like sparse coding and dictionary learning algorithms were utilized for the automated grading of nuclear pleomorphism. The joint kernel-based supervised hashing approach was proposed by Jiang et al. [119]. The proposed approach integrates complementary features in a hashing framework. Classification accuracy of 91% within 16.5ms query time was reported.

Beck et al. [120] designed a model namely the C-Path system to measure the quantitative features from epithelial and stromal regions of breast cancer tissues. Standard and morphometric descriptors of image objects were considered. The features such as higher-level contextual features and global image features were considered. Baker et al. [121] proposed a framework for the classification of BCHI using a combination of K-means clustering and watershed algorithms for the segmentation. Morphology features were extracted from the segmented ROI. An accuracy of 70% and 86% were reported by rule-based and decision tree classifiers.

Irshad et al. [122] developed an automated mitotic detection framework based on different texture features. Texture features such as co-occurrence features, run-length features, and scale-invariant feature transform were extracted and used for the classification. A method for counting the mitotic cells in histopathological images was proposed in [123]. The intensity-based and Haralick features were extracted from the mitotic nuclei and surrounding stromal regions. Regenerative random forest classifier was utilized to classify the mitotic and non-mitotic nuclei. Nateghi et al. [124] reported a method to remove the non-mitotic cells from BCHI by using maximized intra-class weighted mean. The color, texture, and shape-based features were extracted. The classification of mitosis and non-mitosis was performed by using SVM with RBF kernel.

#### Classification using DL approach

Several studies have been carried out to classify BCHI into benign and malignant using DL techniques [125–131]. The features were extracted by using VGG16, VGG17, DenseNet, ResNet, InceptionV3, and AlexNet architectures and were classified using dense layers.

Fully Convolutional Neural Network (FCNN) architecture for detection and classification using 240 WSIs of the breast was proposed by Gece et al. [132]. DCNN architecture for classification of BCHI was developed by Burçak et al. [133]. A model was designed to compute weights in the network and update the parameters for faster backpropagation learning. A class structure-based DCNN architecture was developed by Han et al. [134] for multi- class classification of BCHI. An average accuracy of 93% was reported on BreakHis dataset. Nucleus guided feature extraction framework was proposed by Zheng et al. [135]. A CNN classifier for detecting the presence of invasive breast cancer from WSIs was proposed by Cruz-Roa et al. [136]. The model was trained using 400 images and validated on 200 cases from the Cancer Genome Atlas dataset.

BreakNet architecture for classification was developed by Togacar et al. [137]. The architecture is a combination of attention modules and hypercolumn techniques. The architecture consists of convolutional, dense, and residual blocks. The classification accuracy of 98% was reported on BreakHis dataset. Li et al. [138] proposed a DCNN model to address the issue of class variance and feature extraction from different magnification images. Xception model is used to extract the features. A residual learning-based CNN named ResHist for automatic diagnosis of BCHI was designed by Gour et al. [139]. The developed CNN architecture consists of 152 layers. The ResHist model achieved an accuracy of 84% and an F1-score of 90% for binary classification.

An approach for extracting the multilevel features by integrating CNN and RNN architecture was proposed by Yan et al. [140]. The model achieved an accuracy of 91% for four-class classification. Breast Cancer Histopathology Image Classification Network (BHCNet) was designed by Jiang et al. [141]. The architecture consists of a combination of residual modules and squeeze-and-excitation blocks to reduce overfitting. Classification accuracy ranging from 98-99% for binary classification and 90-93% for multi-class classification was reported. Transfer learning approach was utilized [142] for detection and classification of breast cancer. To extract local information from the images and to improve the classification accuracy, Interleaved DenseNet with SENet architecture was proposed by Li et al. [143]. Performance of the transfer learning techniques for classification of epithelial and stromal regions was investigated by Du et al. [144].

Wang et al. [145] proposed a hybrid structure for the classification of BCHI. The high-level feature was extracted by incorporating transfer learning and double-deep transfer learning techniques. The interactive approach was used to enhance the classification performance. The classification accuracy ranging from 96-98% was reported. Context-aware stacked CNN for analyzing a large contextual area in WSIs for classification was reported by Bejnordi et al. [146]. Inception_ResNet_V2 architecture [147] was used to classify BCHI. The pre-trained architectures namely ResNet50 and DenseNet-161 were used to extract the features and to detect IDC by Celik et al. [148]. The experiment was conducted on 277,524 image patches of 50X50 pixels. An accuracy of 91% was reported on DenseNet-161 and 94% on ResNet-50 respectively.

Sharma and Mehra [149] demonstrated the influence of layer-wise fine-tuning for the classification of images using a pertained network. The experiment was carried out using per-trained AlexNet architecture with 8 layers. From this study, the authors claimed that moderate level of fine-tuning is an ideal choice for classification. Alzubaidi et al. [150] utilized transfer learning technique to handle the inadequacy in training datasets. Hybrid DCNN architecture with parallel convolutional layers with multi-branch and residual links was designed. A selective attention mechanism was designed by Xu et al. [151] to identify potential regions from the BCHI. Haar wavelet-based spectral features were integrated with spatial features to reinforce the performance of CNN [152].

Authors in [153] implemented an ensemble of multiscale CNN architecture for the classification. The scaled images were used to fine-tune the pre-trained architectures namely DenseNet-161, ResNet-152, and ResNet-101. The authors also claimed that the proposed architecture reduces the time complexity with an accuracy of 91% on training datasets. Kausar et al. [154] proposed a method for image classification using CNN on wavelet decomposed images. An accuracy of 98% was reported on International Conference on Image Analysis and Recognition (ICIAR) datasets. Yang et al. [155] designed an architecture to focus the network only on specific regions of interest by incorporating a guided soft attention network. A patch-based classifier was developed by Roy et al. [156] by utilizing CNN for automation. The proposed method was tested on the ICIAR-2018 dataset. An accuracy ranging from 77-92% was reported.

To overcome the computational cost concerning very large images Nazeri et al. [157] proposed a two-stage CNN architecture. The “patch-wise” network is designed to extract the local information from the image patches. The “image-wise” network was designed to extract global information and to perform classification. An accuracy of 95% was reported on the validation sets. Bejnordi et al. [158] designed CNN architecture to identify and distinguish tumor-associated stromal regions in breast biopsies. A transfer learning approach with block-wise fine-tuning was utilized to learn the best features from the images to handle magnification dependent and magnification independent binary and eight class classification problems [159]. CNN-based approach was designed to classify images based on nuclear atypia grading [160]. Multi-scale feature concatenation for the classification of BCHI was carried out by Kausar et al. [161] using DCNN. Bi-directional Long-Short Term Memory model [162] approach was also proposed for the classification using context-based patch modeling. A deep transfer network [130] using Deep Convolutional Generative Adversarial Network (DCGAN) as a data augmentation technique was proposed to tackle the data imbalance problem.

Many studies have been carried out on the classification of mitotic and non-mitotic nuclei using DL techniques [163–166]. Multi-stage mitotic cell detection methods based on faster region CNN and deep CNNs was proposed by Mahmood et al. [167]. Resnet-50 network was utilized for feature extraction. Wu et al. [168] developed a fused FCNN architecture by combining the features from various layers to detect mitosis. The method was validated on the 2014 ICPR MITOSIS dataset. A two-phase CNN to reduce the class imbalance problem while classifying mitotic and non-mitotic nuclei was reported by [169].

#### Classification using hybrid approach

Various approaches have been proposed for the classification of BCHI using hybrid techniques [29, 170, 170] .Usually, automated feature engineering is used in the case of CNN. However, in a hybrid approach, handcrafted features are extracted and classified using neural network. CNN [29] architecture was designed to extract the features at different scales in BCHI. SVM classifier was used to classify the images. A sensitivity of 95% and an accuracy of 83% were reported. Wan et al. [170] reported a cascading ensemble approach for grading the BCHI. Multi-level features and semantic level features derived from CNN were extracted and trained an SVM classifier. The study was performed on 106 biopsy slides. Shallu et al. [171] utilized a pre-trained network namely VGG16, VGG19, and ResNet50 for feature extraction. An accuracy of 92% with logistic regression classifier for VGG16 was reported.

Comparison of two ML approaches for the classification was studied by [172]. Handcrafted features were extracted and trained on SVM and CNN classifier for classification. They reported an accuracy ranging from 96-98% for binary classification and 83-88% for multi-class classification. DNN models guided by the clustered algorithms to identify the hidden structural and statistical information from images were developed by Nahid et al. [173]. An accuracy of 91% on 200X and a precision of 96% on 40X images were reported. George et. al [174] proposed a nucleus-guided transfer learning approach for BCHI. The features were extracted using CNN pre-trained on ImageNet. SVM classifier was utilized to perform the classification of fused features. Outputs of the CNN and SVM were combined by using the belief theory-based classifier fusion technique. An accuracy of 96%, a sensitivity of 97%, and specificity of 96% were reported.

An analysis on cellularity estimation in BCHI was studied by Pei et al. [175]. The cellularity estimation was carried out by combining deep features, SVM, and tree boosting. The authors in [176] developed a predictive algorithm for the automation of benign and malignant proliferative breast lesions. The classifiers consist of two regression-based, two DL based, and two tree-based learning algorithms. The combination of a logistic regression model with active feature extraction outperformed other models with an accuracy of 91%.

A multi-network feature extraction model was developed by Wang et al. [177]. The features were extracted by utilizing four pre-trained DCNNs namely DenseNet-121, ResNet-50, Multi-level InceptionV3, and multi-level VGG-16. The relevant features were selected by the dual-network orthogonal low-rank learning technique. Classification was performed by using Ensemble_SVM classifier. The experiment was evaluated on ICIAR 2018 dataset and classification accuracy of 97% was reported. Incremental boosting CNN was designed [178] for classification. Global and local features were extracted by an ensemble of DCNNs from multi-scale images. The gradient boosting tree approach was utilized to perform the classification. Sharma and Mehra [179] explored and compared two ML techniques for the classification of BCHI. Handcrafted features namely Hu moment, color histogram, and Haralick textures were extracted and trained on conventional classifiers. In the second approach, pre-existing architectures were utilized to extract the features. Their study revealed that the use of transfer learning approaches for feature extraction gives better results when compared with handcrafted approaches. The authors in [180] explored ten pre-trained CNNs for feature extraction. The extracted features were classified using SVM classifiers. The feature extractor architectures namely ResNet 50, ResNet 101 and AlexNet with SVM classifiers were giving a better detection rate.

The cascading approach to detect mitotic nuclei was proposed by [181]. Handcrafted features such as morphology, intensity, and texture features along with CNN features were extracted and combined to detect the mitotic nuclei. The proposed method reported an F-measure of 0.73. Saha et al. [182] designed a DL architecture with handcrafted features to detect mitotic cells from BCHI. The architecture comprises five convolution layers, four max-pooling layers, four ReLU, and two fully connected layers. The morphology, texture, and intensity features were extracted. They reported a precision, recall, and F1-score of 92%, 88%, and 90% respectively. Beevi et al. [183] reported a method to detect mitosis using transfer learning technique. The features were extracted by combining a pre-trained CNN with a random trees classifier. An F1-score of 94% was reported. Mask-RCNN architecture was utilized to classify the mitotic and non-mitotic cells [184]. The handcrafted features namely shape and texture features are extracted from the image. A precision, recall, and F1-score of 93%, 81%, and 0.87 on the ICPR 2012 dataset was reported.

**Fig. 9:**
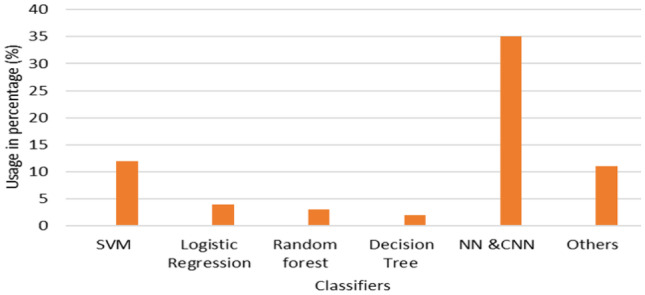
Illustration of various classifiers used in the literature

Figure 9 shows the illustration of various classification approaches used in the literature. We grouped them into traditional, DL, and hybrid approaches. In the “others” category we included K-NN, rule-based classifiers, boosting tree classifier, random subspace ensemble and belief theory-based classifiers. A summary of state-of-the-art approaches is given in Table 3.

**Table 3:** Summary of the state-of-the-art approaches

**Year**	**Pre-processing**	**Segmentation**	**Feature Extraction**	**Classification**	**Evaluation Metrix**	**Ref**
*2016*	NP	NP	Curvelet, LBP	SVM, Random forest, Decision tree, Polynomial classifiers	Acc=91% (Polynomial classifier)	[115]
*2017*	Color deconvolution	NP	LBP	Random Decision Tree	Acc=84%	[113]
*2017*	Macenko, Nonlinear transformation	Thresholding	Color, texture, Shape	SVM	F-score=88%	[185]
*2017*	Non liner mapping	Hybrid active counter	Pixel, Object, semantic level	SVM	Acc=92%	[170]
*2017*	Macenko	NP	Color, shape, Nuclear density	CNN, SVM	Sen=95%	[29]
*2018*	Macenko	NP	CNN	FCN	Acc=87%	[156]
*2018*	Gaussian Blur Filters	K-means, Watershed	Morphology, Geometric	Rule-based, Decision Tree	Acc=70-86%	[121]
*2019*	Macenko	NP	VGG16	FCN	Acc=94-97%	[161]
*2019*	Color deconvolution	NP	VGGNet	Random forest, FCN	Sen=90%, Pre=87%, F1-score=88%	[183]
*2019*	Macenko	NP	Inception network	Gradient Boosting Tree	Acc=91-95% [BreakHis]	[178]
*2019*	Quantile normalization	Hybrid level set	CNN	SVM	Acc=90%	[56]
*2019*	Macenko	NP	GoogleNet, VGGNet, ResNet	FCN	Acc=97%	[142]
*2020*	Image rescaling	NP	VGG16, VGG19, Xception, ResNet50	SVM, Logistic regression	Acc=83-93%	[129]
*2020*	Macenko	Laplacian of Gaussian	AlexNet, ResNet-18, ResNet50, ResNet-101, GoogleNet	SVM	Acc=96%, Sen=97%	[174]
*2020*	Color enhancement	NP	ResNet-50, DenseNet-121, ML-InceptionV3, ML-VGG16	E-SVM	Acc=97%	[177]
*2020*	NP	NP	ResNet50, DenseNet-161	FCN	Acc=91%	[148]

NP=Not performed; ACC=Accuracy; Sen=Sensitivity; Pre=Precision

## Discussion

In recent years, BCHI analysis using medical image processing techniques has gained much popularity among the research community. Manual analysis of BCHI is prone to observer variability, human errors and is a tedious process. To mitigate these challenges, the use of CAD systems for the diagnosis of breast cancer using histopathological images is considered a potential alternative. However, there are challenges in developing CAD systems which are summarized below along with future directions.

### Dataset

**Fig. 10:**
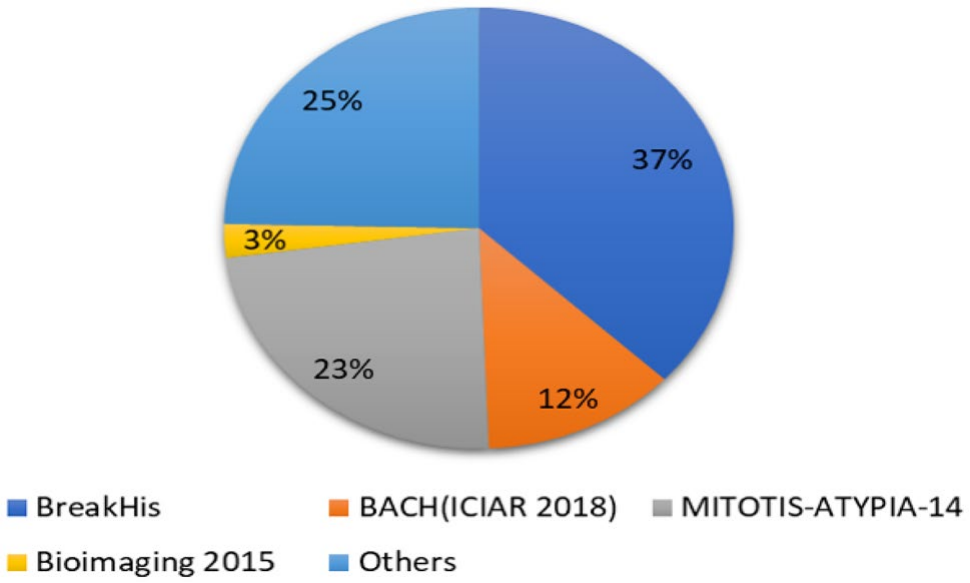
Illustration of the database used in the literature

The development of CAD systems for analysis of BCHI is greatly dependent on the availability of large-scale datasets with annotations. Moreover, different annotations need to be provided for semantic segmentation and classification. For example, segmentation of nuclei needs annotations where nuclei regions are marked, image-level annotations are necessary for the image classification problem, bounding box based annotations are needed for detection problems. However, providing these annotations requires domain knowledge and is a tedious task. Considerable care must be taken while providing annotations since it has a great impact on the learning algorithm. The lack of a standard dataset in literature for the analysis of BCHI limits the development of CAD systems. In our view, the establishment of a standard large-scale dataset with annotations is the need of the hour as it provides a common platform to compare various algorithms. Summary of the most frequently used datasets in the literature is shown in Fig. 10. From the literature, it can be observed many research groups used BreakHis and Bioimaging 2015 database images for their study.

**Fig. 11:**
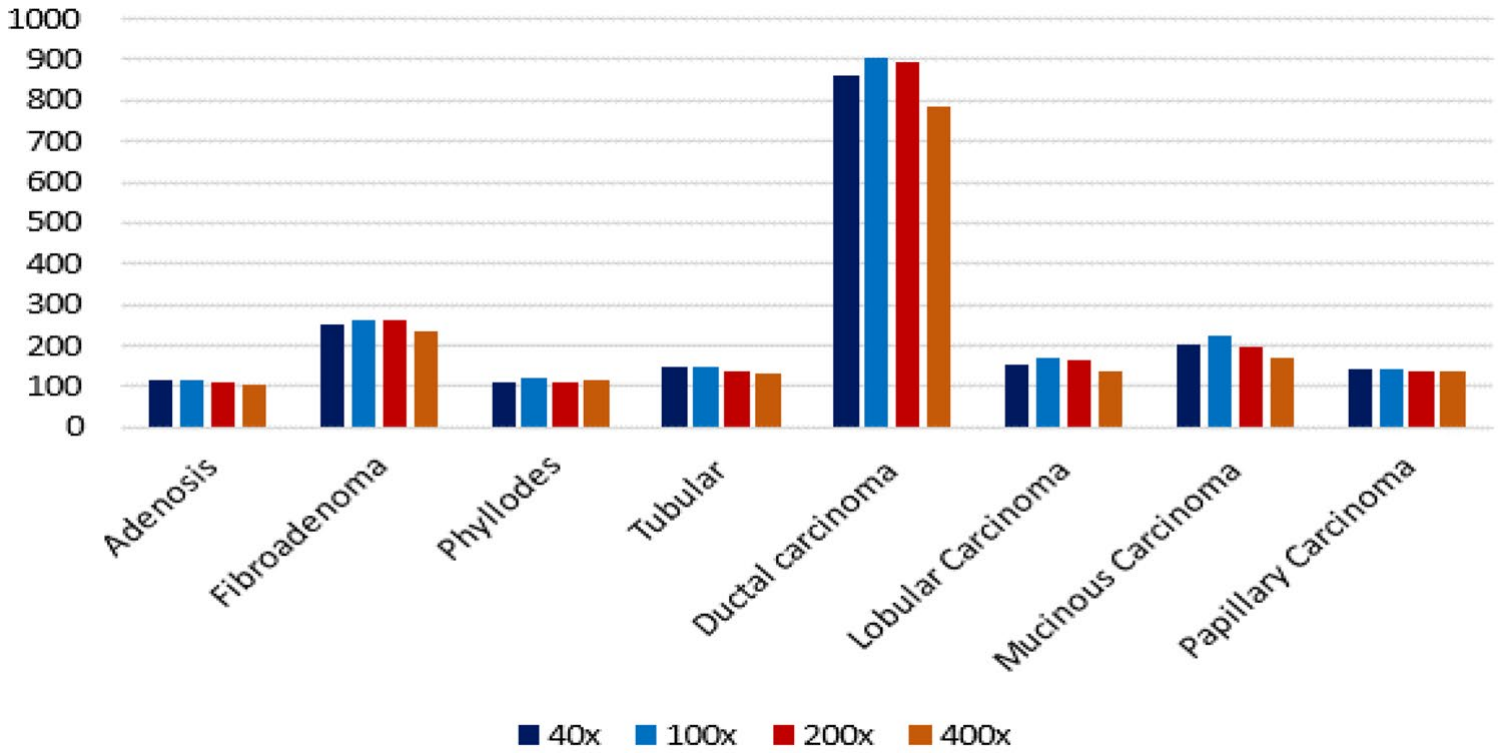
Distribution of image samples for different categories of diseases in BreakHis dataset

**Fig. 12:**
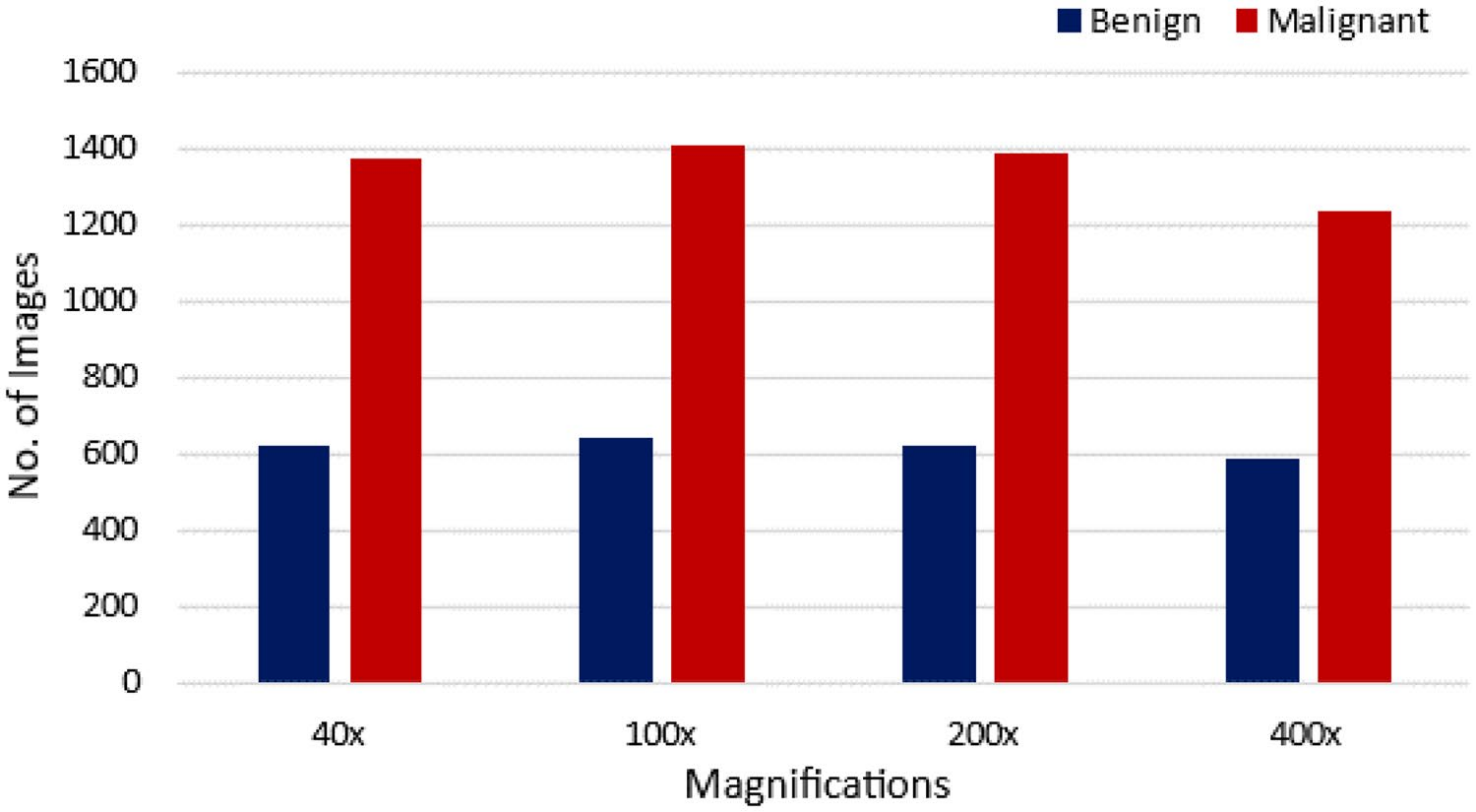
Distribution of image samples for benign and malignant cases in BreakHis dataset

One of the most popular publicly available database for the analysis of BCHI is BreakHis [24]. From Figures 11 and 12 we can see that the number of cases for the benign class is significantly less as compared to the malignant class. This imbalance in classes results in poor training of DL models. The class imbalance problem is one of the challenges which needs to be addressed. A possible solution is to consider data augmentation to increase the number of samples for the benign class.

### Magnification factors

To determine the condition of tumor, a pathologist analyses the images at varying magnifications such as 40x, 100x, 200x, and 400x. At 100x magnification, nuclei distribution and tissue structures are studied which helps in the determination of different types of cancer. At 400x magnification, various characteristics of nuclei are studied namely mitotic count, prominent nuclei and hyperchromatic nuclei. Hence, each magnification plays an important role in the determination of breast cancer. Despite this interest, most researchers have focused on developing CAD systems for 400x magnification images which may fail to capture the distribution of the tissue structure. It can be thus suggested that, in the future more attention could be given to the analysis of 100x and 200x magnification images for the determination of breast cancer.

### Color normalization

The slide preparation process has a large impact on the results since variations in the slide preparation process result in different color distribution of the histopathological images. To handle variations in color distribution, the color normalization process is popularly used. It is observed in the literature that there are no appropriate evaluation metrics for evaluating color normalization processes. Hence, identifying the proper metrics for evaluation of the color normalization process is important. Also, GAN can be explored in the future as it can learn to generate images based on constraints and hence, can be used to transfer the color distribution of a reference image to an input image.

### ROI segmentation

The most prominent task in the analysis of BCHI is studying the structure and characteristics of nuclei since it contributes greatly to the determination of malignancy of the tumor. This involves the segmentation of nuclei which is challenging due to clustered and overlapping nuclei, heterogeneous structure of nuclei and poor staining process. Moreover, at 100x magnification, nuclei are small in size. Several algorithms are developed in literature based on traditional and DL based methods to segment nuclei. Watershed-based algorithm is popularly used to separate connected nuclei. However, the separation of overlapping nuclei is seldom addressed in the literature. Also, the segmentation of nuclei from 100x magnification is very challenging as it needs annotations at 100x magnification for the validation process which is a tedious process.

### Traditional methods vs DL

Earlier traditional methods which include the pipelined approach had much popularity in processing BCHI. These methods include various steps such as pre-processing, segmentation, feature extraction, and classification. However, the accuracy of these systems depends on the segmentation process and feature extraction from the identified regions. Handcrafted features were used to define the ROI. However, the handcrafted features may fail to capture all the variations in the pattern of the data and thus reducing the accuracy of the system. Also, developing these systems required extensive domain knowledge in image processing and histopathological images.

**Fig. 13:**
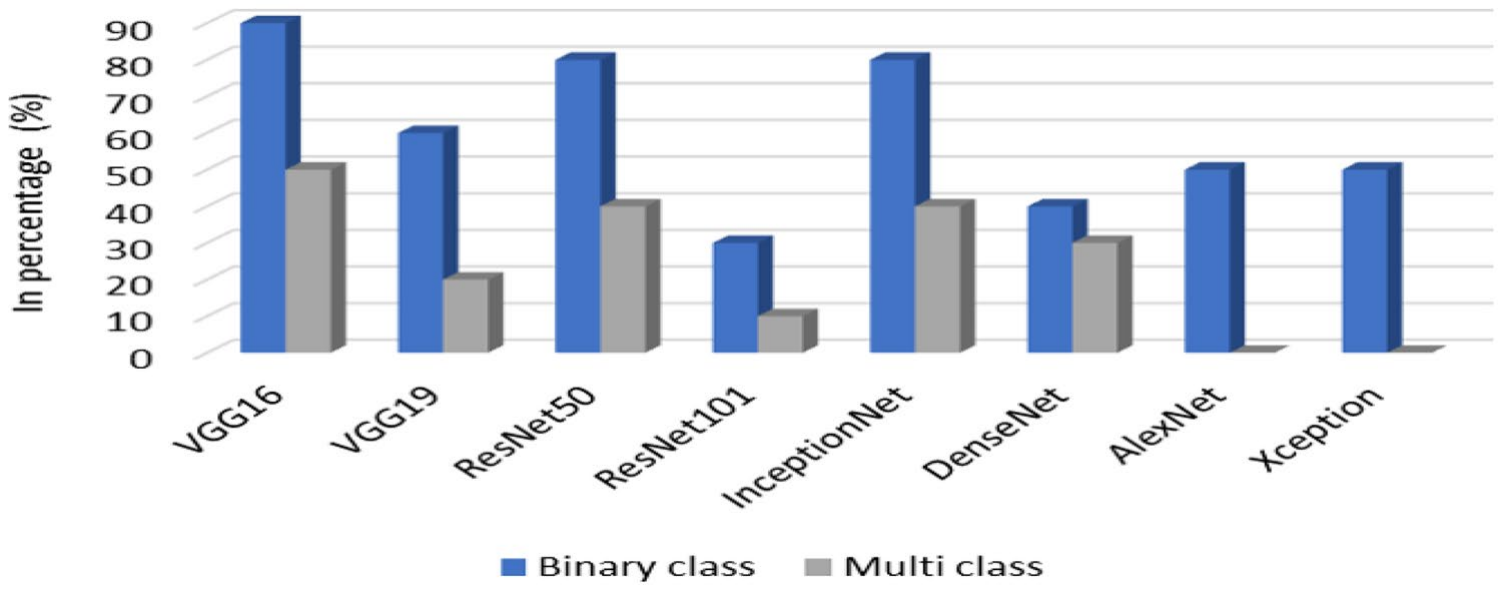
Description of various CNN architectures used for binary and multi-class classification

DL has gained much popularity in the last few years for processing of histopathological images due to its ability to model complex patterns and increases the computational power [14, 186]. CNN is a popular choice for feature extraction as it learns to extract the most relevant features based on the backpropagation algorithm. However, researchers have to analyze the results of all layers to justify the performance of the developed model for the analysis of BCHI. Also, the development of CNN architecture for the analysis of histopathological images requires expertise in DL. But DL-based algorithms require a large-scale dataset with annotation for training the models. The lack of standard datasets with annotations makes it challenging to develop DL-based models for processing histopathological images. Moreover, feature visualization and analysis is necessary to understand the behaviour of the model. Figure 13 shows the usage of various popular CNN architectures used for multiclass and binary classification problems. It is observed that VGG-16, ResNet-50, and InceptionNet are popular choices for binary classification problems. VGG-16 and ResNet-50 are also used for multiclass classification of BCHI. Also, it is observed that deeper architectures such as VGG-19 and ResNet101 are less explored for classification as compared to other methods.

## Challenges

There is a considerable amount of literature on the application of medical image processing techniques to process BCHI. However, there exists a few challenges which are given below.

- The lack of standard datasets makes it difficult to evaluate and compare various methods. A standard dataset would provide various researchers a common platform facilitating appropriate comparison.
- Creating annotations for nuclei segmentation is tedious, time consuming and challenging.
- Segmentation of nuclei from 400x magnification is still a challenge due to overlapping and clustered nuclei. Further, segmentation of nuclei at 100x is challenging due to the small size, varying structure, and random distributions of nuclei.
- There are no standard metrics to evaluate the performance of the color normalization methods.
- There is scope for developing a unified algorithm for the segmentation of nuclei and classification of histopathological images at varying magnifications holistically.
- The heterogeneous characteristics of malignant samples make it difficult to model the patterns to differentiate them from benign samples.
- CNN based methods for histopathological image classification extracts features from the entire image and may fail to focus on the regions of interest such as nuclei, gland and mitotic cells, which contribute largely to the decision of classifying images as malignant and benign. Hence, there is scope for incorporating attention mechanism in CNN to enable the model to focus on a potential ROI.

## Conclusion

This paper provided a comprehensive overview of the state-of-the-art literature in the area of BCHI analysis for diagnostic purposes using image processing techniques. We noticed that a lot of efforts have been made to develop methods for the automation of segmentation, detection of specific ROIs, and classification of images into benign and malignant and into their subclasses. We have summarized various state-of-the-art methods for processing, segmentation, and classification of BCHI. The lack of a standard dataset with annotations is the main hindrance to the development of CAD systems. There exist a few other challenges in the processing of BCHI that need to be addressed such as overlapping and clustered nuclei, heterogeneous structure of nuclei etc. DL-based algorithms are the future of BCHI processing. Moreover, the research could focus on processing BCHI at various magnifications such as 40x, 100x, 200x, and 400x together as it helps in better analysis of different subtypes of breast cancer. We presented a methodological review of the various algorithms used for the development of CAD systems for BCHIs. We also highlighted the general procedures for the analysis of BCHI along with various challenges. This can act as a starting point for new researchers to work on the development of CAD systems for breast cancer detection.

## Abbreviations

BCHI: Breast Cancer Histopathological Image
CAD: Computer-Aided Diagnosis System
WSI: Whole Slide Images
ROI: Region Of Interest
ML: Machine Learning
DL: Deep Learning
H[MYAMP: E] Hematoxylin and Eosin
IDC: Invasive Ductal Carcinoma
BACH: BreAst Cancer Histology
ICIAR: International Conference on Image Analysis and Recognition
BreCaHAD: Breast Cancer Histopathological Annotation and Diagnosis
DCNN: Deep Convolutional Neural Network
CNN: Convolutional Neural Network
RCNN: Recurrent Convolutional Neural Network
LBP: Local Binary Pattern
SVM: Support Vector Machine
RBF: Radial Basis Function kernel
FCNN: Fully Convolutional Neural Network
GAN: Generative Adversarial Network
VGG: Visual Geometry Group
ResNet: Residual Neural Network
DCGAN: Deep convolutional Generative Adversarial Network

## References

[1] Siegel, R.L., Miller, K.D., Jemal, A.: Cancer statistics, 2019. CA: A Cancer Journal for Clinicians **69**(1), 7–34 (2019) 10.3322/caac.21551 30620402

[2] Bray, F., Ferlay, J., Soerjomataram, I., Siegel, R.L., Torre, L.A., Jemal, A.: Global cancer statistics 2018: GLOBOCAN estimates of incidence and mortality worldwide for 36 cancers in 185 countries. CA: A Cancer Journal for Clinicians **68**(6), 394–424 (2018) 10.3322/caac.21492 30207593

[3] Ferlay J, Colombet M, Soerjomataram I, Mathers C, Parkin D, Piñeros M, Znaor A, Bray F (2019). Estimating the global cancer incidence and mortality in 2018: GLOBOCAN sources and methods. International Journal of Cancer.

[4] Ghoncheh M, Pournamdar Z, Salehiniya H (2016). Incidence and mortality and epidemiology of breast cancer in the world. Asian Pacific Journal of Cancer Prevention.

[5] Kumar, V., Abbas, A.K., Aster, J.C.: Robbins basic pathology E-book. Elsevier Health Sciences (2017)

[6] Mills, S.E., Carter, D., Greenson, J.K., Reuter, V.E., Stoler, M.H.: Sternberg’s diagnostic surgical pathology. Lippincott Williams & Wilkins (2012)

[7] He L, Long LR, Antani S, Thoma GR (2012). Histology image analysis for carcinoma detection and grading. Computer Methods and Programs in Biomedicine.

[8] Demir C, Yener B (2005). Automated cancer diagnosis based on histopathological images: a systematic survey.

[9] Young, B., Woodford, P., O’Dowd, G.: Wheater’s Functional Histology E-Book: A Text and Colour Atlas. Elsevier Health Sciences (2013)

[10] Mohan, H.: Textbook of Pathology, Harsh Mohan, 2010, Jaypee Brothers Medical Publishers(P) ltd. Jaypee Brothers Medical Publishers (P) Ltd (2010)

[11] Gurcan MN, Boucheron LE, Can A, Madabhushi A, Rajpoot NM, Yener B (2009). Histopathological image analysis: A review. IEEE Reviews in Biomedical Engineering.

[12] Veta M, Pluim JP, Van Diest PJ, Viergever MA (2014). Breast cancer histopathology image analysis: A review. IEEE Transactions on Biomedical Engineering.

[13] Ghaznavi F, Evans A, Madabhushi A, Feldman M (2013). Digital imaging in pathology: whole-slide imaging and beyond. Annual Review of Pathology: Mechanisms of Disease.

[14] Holzinger, A., Goebel, R., Mengel, M., Müller, H.: Artificial Intelligence and Machine Learning for Digital Pathology: State-of-the-art and Future Challenges, vol. 12090. Springer Nature (2020)

[15] Krenacs, T., Zsakovics, I., Micsik, T., Fonyad, L., Varga, S.V., Ficsor, L., Kiszler, G., Molnar, B.: Digital microscopy: the upcoming revolution in histopathology teaching, diagnostics, research and quality assurance. Microscopy: Science, Technology, Applications and Education **2**, 965–977 (2010)

[16] Irshad H, Veillard A, Roux L, Racoceanu D (2013). Methods for nuclei detection, segmentation, and classification in digital histopathology: a review-current status and future potential. IEEE Reviews in Biomedical Engineering.

[17] Madabhushi A, Lee G (2016). Image analysis and machine learning in digital pathology: Challenges and opportunities. Medical Image Analysis.

[18] Furat NT, Alwan NA, Khashman BM (2018). Optimization of digital histopathology image quality. IAES International Journal of Artificial Intelligence.

[19] Komura D, Ishikawa S (2018). Machine learning methods for histopathological image analysis. Computational and Structural Biotechnology Journal.

[20] Nahid, A.A., Kong, Y.: Involvement of machine learning for breast cancer image classification: a survey. Computational and Mathematical Methods in Medicine **2017** (2017) 10.1155/2017/3781951 PMC5804413 29463985

[21] Yassin NI, Omran S, El Houby EM, Allam H (2018). Machine learning techniques for breast cancer computer aided diagnosis using different image modalities: A systematic review. Computer Methods and Programs in Biomedicine.

[22] Onder D, Zengin S, Sarioglu S (2014). A review on color normalization and color deconvolution methods in histopathology. Applied Immunohistochemistry & Molecular Morphology.

[23] Roy, S., kumar Jain, A., Lal, S., Kini, J.: A study about color normalization methods for histopathology images. Micron **114**, 42–61 (2018) 10.1016/j.micron.2018.07.005 30096632

[24] Spanhol FA, Oliveira LS, Petitjean C, Heutte L (2015). A dataset for breast cancer histopathological image classification. IEEE Transactions on Biomedical Engineering.

[25] Aresta G, Araújo T, Kwok S, Chennamsetty SS, Safwan M, Alex V, Marami B, Prastawa M, Chan M, Donovan M (2019). BACH: Grand challenge on breast cancer histology images. Medical Image Analysis.

[26] Mitos-atypia-14. https://mitos-atypia-14.grand-challenge.org/dataset/ (Aug 2020)

[27] Veta M, Heng YJ, Stathonikos N, Bejnordi BE, Beca F, Wollmann T, Rohr K, Shah MA, Wang D, Rousson M (2019). Predicting breast tumor proliferation from whole-slide images: the TUPAC16 challenge. Medical Image Analysis.

[28] Camelyon16. https://camelyon16.grand-challenge.org/data/ (Aug 2020)

[29] Araújo T, Aresta G, Castro E, Rouco J, Aguiar P, Eloy C, Polónia A, Campilho A (2017). Classification of breast cancer histology images using convolutional neural networks. Plos One.

[30] Invasive Ductal Carcinoma (IDC) Histology Image Dataset. http://www.andrewjanowczyk.com/use-case-6-invasive-ductal-carcinoma-idc-segmentation/ (Jan 2020)

[31] Camelyon17. https://camelyon17.grand-challenge.org (Aug 2020)

[32] Aksac A, Demetrick DJ, Ozyer T, Alhajj R (2019). BreCaHAD: a dataset for breast cancer histopathological annotation and diagnosis. BMC Research Notes.

[33] Amgad M, Elfandy H, Hussein H, Atteya LA, Elsebaie MA, Abo Elnasr LS, Sakr RA, Salem HS, Ismail AF, Saad AM (2019). Structured crowdsourcing enables convolutional segmentation of histology images. Bioinformatics.

[34] Amgad, M., Atteya, L.A., Hussein, H., Mohammed, K.H., Hafiz, E., Elsebaie, M.A., Alhusseiny, A.M., AlMoslemany, M.A., Elmatboly, A.M., Pappalardo, P.A., et al.: Nucls: A scalable crowdsourcing, deep learning approach and dataset for nucleus classification, localization and segmentation. arXiv preprint arXiv:2102.09099 (2021) 10.1093/gigascience/giac037 PMC9112766 35579553

[35] Pan X, Lu Y, Lan R, Liu Z, Qin Z, Wang H, Liu Z (2021). Mitosis detection techniques in h&e stained breast cancer pathological images: A comprehensive review. Computers & Electrical Engineering.

[36] Janowczyk, A., Madabhushi, A.: Deep learning for digital pathology image analysis: A comprehensive tutorial with selected use cases. Journal of Pathology Informatics **7** (2016) 10.4103/2153-3539.186902 PMC4977982 27563488

[37] Loukas CG, Linney A (2004). A survey on histological image analysis-based assessment of three major biological factors influencing radiotherapy: proliferation, hypoxia and vasculature. Computer Methods and Programs in Biomedicine.

[38] Fuchs TJ, Buhmann JM (2011). Computational pathology: challenges and promises for tissue analysis. Computerized Medical Imaging and Graphics.

[39] Zhang, S., Metaxas, D.: Large-scale medical image analytics: Recent methodologies, applications and future directions (2016) 10.1016/j.media.2016.06.010 27503077

[40] Litjens G, Kooi T, Bejnordi BE, Setio AAA, Ciompi F, Ghafoorian M, Van Der Laak JA, Van Ginneken B, Sánchez CI (2017). A survey on deep learning in medical image analysis. Medical Image Analysis.

[41] Benhammou Y, Achchab B, Herrera F, Tabik S (2020). Breakhis based breast cancer automatic diagnosis using deep learning: Taxonomy, survey and insights. Neurocomputing.

[42] Steiner DF, MacDonald R, Liu Y, Truszkowski P, Hipp JD, Gammage C, Thng F, Peng L, Stumpe MC (2018). Impact of deep learning assistance on the histopathologic review of lymph nodes for metastatic breast cancer. The American Journal of Surgical Pathology.

[43] Debelee TG, Schwenker F, Ibenthal A, Yohannes D (2020). Survey of deep learning in breast cancer image analysis. Evolving Systems.

[44] Srinidhi, C.L., Ciga, O., Martel, A.L.: Deep neural network models for computational histopathology: A survey. Medical Image Analysis p. 101813 (2020) 10.1016/j.media.2020.101813 PMC7725956 33049577

[45] Chugh, G., Kumar, S., Singh, N.: Survey on machine learning and deep learning applications in breast cancer diagnosis. Cognitive Computation pp. 1–20 (2021)

[46] Bejnordi, B.E., Timofeeva, N., Otte-Höller, I., Karssemeijer, N., van der Laak, J.A.: Quantitative analysis of stain variability in histology slides and an algorithm for standardization. In: Medical Imaging 2014: Digital Pathology. vol. 9041, p. 904108. International Society for Optics and Photonics (2014)

[47] Tosta, T.A.A., de Faria, P.R., Neves, L.A., do Nascimento, M.Z.: Computational normalization of h&e-stained histological images: Progress, challenges and future potential. Artificial Intelligence in Medicine **95**, 118–132 (2019) 10.1016/j.artmed.2018.10.004 30420242

[48] Basavanhally, A., Madabhushi, A.: EM-based segmentation-driven color standardization of digitized histopathology. In: Medical Imaging 2013: Digital Pathology. vol. 8676, p. 86760G. International Society for Optics and Photonics (2013)

[49] Li X, Plataniotis KN (2015). A complete color normalization approach to histopathology images using color cues computed from saturation-weighted statistics. IEEE Transactions on Biomedical Engineering.

[50] Roy S, Lal S, Kini JR (2019). Novel color normalization method for Hematoxylin & Eosin stained histopathology images. IEEE Access.

[51] Vahadane A, Peng T, Sethi A, Albarqouni S, Wang L, Baust M, Steiger K, Schlitter AM, Esposito I, Navab N (2016). Structure-preserving color normalization and sparse stain separation for histological images. IEEE Transactions on Medical Imaging.

[52] Macenko, M., Niethammer, M., Marron, J.S., Borland, D., Woosley, J.T., Guan, X., Schmitt, C., Thomas, N.E.: A method for normalizing histology slides for quantitative analysis. In: 2009 IEEE International Symposium on Biomedical Imaging: From Nano to Macro. pp. 1107–1110. IEEE (2009)

[53] Tosta, T.A.A., de Faria, P.R., Neves, L.A., do Nascimento, M.Z.: Color normalization of faded H&E-stained histological images using spectral matching. Computers in Biology and Medicine **111**, 103344 (2019) 10.1016/j.compbiomed.2019.103344 31279982

[54] Zarella MD, Yeoh C, Breen DE, Garcia FU (2017). An alternative reference space for H&E color normalization. Plos One.

[55] Reinhard E, Adhikhmin M, Gooch B, Shirley P (2001). Color transfer between images. IEEE Computer Graphics and Applications.

[56] Cao, J., Qin, Z., Jing, J., Chen, J., Wan, T.: An automatic breast cancer grading method in histopathological images based on pixel-, object-, and semantic-level features. In: 2016 IEEE 13th International Symposium on Biomedical Imaging (ISBI). pp. 1151–1154. IEEE (2016)

[57] Gadermayr, M., Cooper, S.S., Klinkhammer, B., Boor, P., Merhof, D.: A quantitative assessment of image normalization for classifying histopathological tissue of the kidney. In: German Conference on Pattern Recognition. pp. 3–13. Springer (2017)

[58] Khan AM, Rajpoot N, Treanor D, Magee D (2014). A nonlinear mapping approach to stain normalization in digital histopathology images using image-specific color deconvolution. IEEE Transactions on Biomedical Engineering.

[59] Bukenya F (2020). A hybrid approach for stain normalisation in digital histopathological images. Multimedia Tools and Applications.

[60] Alsubaie N, Trahearn N, Raza SEA, Snead D, Rajpoot NM (2017). Stain deconvolution using statistical analysis of multi-resolution stain colour representation. Plos One.

[61] Anghel A, Stanisavljevic M, Andani S, Papandreou N, Rüschoff JH, Wild P, Gabrani M, Pozidis H (2019). A high-performance system for robust stain normalization of whole-slide images in histopathology. Frontiers in Medicine.

[62] Kothari, S., Phan, J.H., Moffitt, R.A., Stokes, T.H., Hassberger, S.E., Chaudry, Q., Young, A.N., Wang, M.D.: Automatic batch-invariant color segmentation of histological cancer images. In: 2011 IEEE International Symposium on Biomedical Imaging: From Nano to Macro. pp. 657–660. IEEE (2011) 10.1109/ISBI.2011.5872492 PMC4983436 27532016

[63] Gupta, V., Singh, A., Sharma, K., Bhavsar, A.: Automated classification for breast cancer histopathology images: Is stain normalization important? In: Computer Assisted and Robotic Endoscopy and Clinical Image-Based Procedures, pp. 160–169. Springer (2017)

[64] Sethi, A., Sha, L., Vahadane, A.R., Deaton, R.J., Kumar, N., Macias, V., Gann, P.H.: Empirical comparison of color normalization methods for epithelial-stromal classification in H and E images. Journal of Pathology Informatics **7** (2016) 10.4103/2153-3539.179984 PMC4837797 27141322

[65] Bejnordi BE, Litjens G, Timofeeva N, Otte-Höller I, Homeyer A, Karssemeijer N, van der Laak JA (2015). Stain specific standardization of whole-slide histopathological images. IEEE Transactions on Medical Imaging.

[66] Stanisavljevic, M., Anghel, A., Papandreou, N., Andani, S., Pati, P., Hendrik Ruschoff, J., Wild, P., Gabrani, M., Pozidis, H.: A fast and scalable pipeline for stain normalization of whole-slide images in histopathology. In: Proceedings of the European Conference on Computer Vision (ECCV) Workshops. pp. 0 (2018)

[67] Magliaro C, Tirella A, Mattei G, Pirone A, Ahluwalia A (2015). HisTOOLogy: an open-source tool for quantitative analysis of histological sections. Journal of Microscopy.

[68] Bautista, P.A., Hashimoto, N., Yagi, Y.: Color standardization in whole slide imaging using a color calibration slide. Journal of Pathology Informatics **5** (2014) 10.4103/2153-3539.126153 PMC3952402 24672739

[69] Ruifrok AC, Johnston DA (2001). Quantification of histochemical staining by color deconvolution. Analytical and Quantitative Cytology and Histology.

[70] Prasad, M.N., Prasad, K., Navya, K.: Color transfer method for efficient enhancement of color images and its application to peripheral blood smear analysis. In: International Conference on Recent Trends in Image Processing and Pattern Recognition. pp. 134–142. Springer (2018)

[71] Clarke EL, Revie C, Brettle D, Shires M, Jackson P, Cochrane R, Wilson R, Mello-Thoms C, Treanor D (2018). Development of a novel tissue-mimicking color calibration slide for digital microscopy. Color Research & Application.

[72] Bautista, P.A., Yagi, Y.: Improving the visualization and detection of tissue folds in whole slide images through color enhancement. Journal of Pathology Informatics **1** (2010) 10.4103/2153-3539.73320 PMC3010592 21221170

[73] Janowczyk A, Basavanhally A, Madabhushi A (2017). Stain normalization using sparse autoencoders (stanosa): application to digital pathology. Computerized Medical Imaging and Graphics.

[74] Zanjani, F.G., Zinger, S., Bejnordi, B.E., van der Laak, J.A., de With, P.H.: Stain normalization of histopathology images using generative adversarial networks. In: 2018 IEEE 15th International Symposium on Biomedical Imaging (ISBI 2018). pp. 573–577. IEEE (2018)

[75] Hamidinekoo, A., Zwiggelaar, R.: Stain colour normalisation to improve mitosis detection on breast histology images. In: Deep Learning in Medical Image Analysis and Multimodal Learning for Clinical Decision Support, pp. 213–221. Springer (2017)

[76] Kowal M, Filipczuk P, Obuchowicz A, Korbicz J, Monczak R (2013). Computer-aided diagnosis of breast cancer based on fine needle biopsy microscopic images. Computers in Biology and Medicine.

[77] Dundar MM, Badve S, Bilgin G, Raykar V, Jain R, Sertel O, Gurcan MN (2011). Computerized classification of intraductal breast lesions using histopathological images. IEEE Transactions on Biomedical Engineering.

[78] Kost, H., Homeyer, A., Bult, P., Balkenhol, M.C., van der Laak, J.A., Hahn, H.K.: A generic nuclei detection method for histopathological breast images. In: Medical Imaging 2016: Digital Pathology. vol. 9791, p. 97911E. International Society for Optics and Photonics (2016)

[79] Veta M, Van Diest PJ, Kornegoor R, Huisman A, Viergever MA, Pluim JP (2013). Automatic nuclei segmentation in h&e stained breast cancer histopathology images. Plos One.

[80] Fatakdawala H, Xu J, Basavanhally A, Bhanot G, Ganesan S, Feldman M, Tomaszewski JE, Madabhushi A (2010). Expectation-maximization-driven geodesic active contour with overlap resolution (emagacor): Application to lymphocyte segmentation on breast cancer histopathology. IEEE Transactions on Biomedical Engineering.

[81] Paramanandam, M., O’Byrne, M., Ghosh, B., Mammen, J.J., Manipadam, M.T., Thamburaj, R., Pakrashi, V.: Automated segmentation of nuclei in breast cancer histopathology images. Plos One **11**(9), e0162053 (2016) 10.1371/journal.pone.0162053 PMC5029866 27649496

[82] Wang P, Hu X, Li Y, Liu Q, Zhu X (2016). Automatic cell nuclei segmentation and classification of breast cancer histopathology images. Signal Processing.

[83] Vink JP, Van Leeuwen M, Van Deurzen C, de Haan G (2013). Efficient nucleus detector in histopathology images. Journal of Microscopy.

[84] Naik, S., Doyle, S., Agner, S., Madabhushi, A., Feldman, M., Tomaszewski, J.: Automated gland and nuclei segmentation for grading of prostate and breast cancer histopathology. In: 2008 5th IEEE International Symposium on Biomedical Imaging: From Nano to Macro. pp. 284–287. IEEE (2008)

[85] Petushi S, Garcia FU, Haber MM, Katsinis C, Tozeren A (2006). Large-scale computations on histology images reveal grade-differentiating parameters for breast cancer. BMC Medical Imaging.

[86] Basavanhally, A., Agner, S., Alexe, G., Bhanot, G., Ganesan, S., Madabhushi, A.: Manifold learning with graph-based features for identifying extent of lymphocytic infiltration from high grade, HER2+ breast cancer histology. Image Anal. Appl. Biol.(in Conjunction MICCAI), New York [Online]. Available: https://engineering.case.edu/centers/ccipd/sites/ccipd.case.edu/files/publications/Manifold-learning-with-graph-based-features-for-identifying-extent-of-lymphocytic-infiltration-from-high-grade-breast-cancer-histology.pdf. (2008)

[87] Kumar A, Prateek M (2020). Localization of nuclei in breast cancer using whole slide imaging system supported by morphological features and shape formulas. Cancer Management and Research.

[88] Bejnordi BE, Balkenhol M, Litjens G, Holland R, Bult P, Karssemeijer N, Van Der Laak JA (2016). Automated detection of DCIS in whole-slide h&e stained breast histopathology images. IEEE Transactions on Medical Imaging.

[89] Salvi M, Molinari F, Dogliani N, Bosco M (2019). Automatic discrimination of neoplastic epithelium and stromal response in breast carcinoma. Computers in Biology and Medicine.

[90] Paul A, Mukherjee DP (2015). Mitosis detection for invasive breast cancer grading in histopathological images. IEEE Transactions on Image Processing.

[91] Maqlin, P., Thamburaj, R., Mammen, J.J., Nagar, A.K.: Automatic detection of tubules in breast histopathological images. In: Proceedings of Seventh International Conference on Bio-Inspired Computing: Theories and Applications (BIC-TA 2012). pp. 311–321. Springer (2013)

[92] Filipczuk P, Fevens T, Krzyżak A, Monczak R (2013). Computer-aided breast cancer diagnosis based on the analysis of cytological images of fine needle biopsies. IEEE Transactions on Medical Imaging.

[93] Wang S, Yang DM, Rong R, Zhan X, Xiao G (2019). Pathology image analysis using segmentation deep learning algorithms. The American Journal of Pathology.

[94] Jung H, Lodhi B, Kang J (2019). An automatic nuclei segmentation method based on deep convolutional neural networks for histopathology images. BMC Biomedical Engineering.

[95] Mehta, S., Mercan, E., Bartlett, J., Weaver, D., Elmore, J., Shapiro, L.: Learning to segment breast biopsy whole slide images. In: 2018 IEEE Winter Conference on Applications of Computer Vision (WACV). pp. 663–672. IEEE (2018)

[96] Naylor, P., Laé, M., Reyal, F., Walter, T.: Nuclei segmentation in histopathology images using deep neural networks. In: 2017 IEEE 14th International Symposium on Biomedical Imaging (ISBI 2017). pp. 933–936. IEEE (2017)

[97] Wang, H., Xian, M., Vakanski, A.: Bending loss regularized network for nuclei segmentation in histopathology images. In: 2020 IEEE 17th International Symposium on Biomedical Imaging (ISBI). pp. 1–5. IEEE (2020) 10.1109/isbi45749.2020.9098611 PMC7733529 33312394

[98] Xing F, Xie Y, Yang L (2015). An automatic learning-based framework for robust nucleus segmentation. IEEE Transactions on Medical Imaging.

[99] Xu J, Xiang L, Liu Q, Gilmore H, Wu J, Tang J, Madabhushi A (2015). Stacked Sparse AutoEncoder (SSAE) for nuclei detection on breast cancer histopathology images. IEEE Transactions on Medical Imaging.

[100] Cruz-Roa, A., Basavanhally, A., González, F., Gilmore, H., Feldman, M., Ganesan, S., Shih, N., Tomaszewski, J., Madabhushi, A.: Automatic detection of invasive ductal carcinoma in whole slide images with convolutional neural networks. In: Medical Imaging 2014: Digital Pathology. vol. 9041, p. 904103. International Society for Optics and Photonics (2014)

[101] Kumar N, Verma R, Sharma S, Bhargava S, Vahadane A, Sethi A (2017). A dataset and a technique for generalized nuclear segmentation for computational pathology. IEEE Transactions on Medical Imaging.

[102] Wan T, Zhao L, Feng H, Li D, Tong C, Qin Z (2020). Robust nuclei segmentation in histopathology using ASPPU-Net and boundary refinement. Neurocomputing.

[103] Xie L, Qi J, Pan L, Wali S (2020). Integrating deep convolutional neural networks with marker-controlled watershed for overlapping nuclei segmentation in histopathology images. Neurocomputing.

[104] Xu J, Gong L, Wang G, Lu C, Gilmore H, Zhang S, Madabhushi A (2019). Convolutional neural network initialized active contour model with adaptive ellipse fitting for nuclear segmentation on breast histopathological images. Journal of Medical Imaging.

[105] Zeng Z, Xie W, Zhang Y, Lu Y (2019). Ric-unet: An improved neural network based on Unet for nuclei segmentation in histology images. IEEE Access.

[106] Mahmood F, Borders D, Chen RJ, McKay GN, Salimian KJ, Baras A, Durr NJ (2019). Deep adversarial training for multi-organ nuclei segmentation in histopathology images. IEEE Transactions on Medical Imaging.

[107] Das DK, Dutta PK (2019). Efficient automated detection of mitotic cells from breast histological images using deep convolution neutral network with wavelet decomposed patches. Computers in Biology and Medicine.

[108] Li C, Wang X, Liu W, Latecki LJ (2018). Deepmitosis: Mitosis detection via deep detection, verification and segmentation networks. Medical Image Analysis.

[109] Sebai M, Wang X, Wang T (2020). Maskmitosis: a deep learning framework for fully supervised, weakly supervised, and unsupervised mitosis detection in histopathology images. Medical & Biological Engineering & Computing.

[110] Wahab N, Khan A, Lee YS (2019). Transfer learning based deep cnn for segmentation and detection of mitoses in breast cancer histopathological images. Microscopy.

[111] Priego-Torres BM, Sanchez-Morillo D, Fernandez-Granero MA, Garcia-Rojo M (2020). Automatic segmentation of whole-slide H&E stained breast histopathology images using a deep convolutional neural network architecture. Expert Systems With Applications.

[112] Bejnordi BE, Veta M, Van Diest PJ, Van Ginneken B, Karssemeijer N, Litjens G, Van Der Laak JA, Hermsen M, Manson QF, Balkenhol M (2017). Diagnostic assessment of deep learning algorithms for detection of lymph node metastases in women with breast cancer. Jama.

[113] Reis S, Gazinska P, Hipwell JH, Mertzanidou T, Naidoo K, Williams N, Pinder S, Hawkes DJ (2017). Automated classification of breast cancer stroma maturity from histological images. IEEE Transactions on Biomedical Engineering.

[114] Zheng Y, Jiang Z, Zhang H, Xie F, Ma Y, Shi H, Zhao Y (2018). Histopathological whole slide image analysis using context-based cbir. IEEE Transactions on Medical Imaging.

[115] Bruno, D.O.T., Do Nascimento, M.Z., Ramos, R.P., Batista, V.R., Neves, L.A., Martins, A.S.: LBP operators on curvelet coefficients as an algorithm to describe texture in breast cancer tissues. Expert Systems with Applications **55**, 329–340 (2016)

[116] Basavanhally AN, Ganesan S, Agner S, Monaco JP, Feldman MD, Tomaszewski JE, Bhanot G, Madabhushi A (2009). Computerized image-based detection and grading of lymphocytic infiltration in her2+ breast cancer histopathology. IEEE Transactions on Biomedical Engineering.

[117] Dimitropoulos K, Barmpoutis P, Zioga C, Kamas A, Patsiaoura K, Grammalidis N (2017). Grading of invasive breast carcinoma through grassmannian VLAD encoding. Plos One.

[118] Das A, Nair MS, Peter SD (2018). Sparse representation over learned dictionaries on the riemannian manifold for automated grading of nuclear pleomorphism in breast cancer. IEEE Transactions on Image Processing.

[119] Jiang M, Zhang S, Huang J, Yang L, Metaxas DN (2016). Scalable histopathological image analysis via supervised hashing with multiple features. Medical Image Analysis.

[120] Beck, A.H., Sangoi, A.R., Leung, S., Marinelli, R.J., Nielsen, T.O., Van De Vijver, M.J., West, R.B., Van De Rijn, M., Koller, D.: Systematic analysis of breast cancer morphology uncovers stromal features associated with survival. Science Translational Medicine **3**(108), 108ra113–108ra113 (2011) 10.1126/scitranslmed.3002564 22072638

[121] Baker, Q.B., Banat, S., Eaydat, E., Alsmirat, M., et al.: Automated detection of benign and malignant in breast histopathology images. In: 2018 IEEE/ACS 15th International Conference on Computer Systems and Applications (AICCSA). pp. 1–5. IEEE (2018)

[122] Irshad, H., Jalali, S., Roux, L., Racoceanu, D., Hwee, L.J., Le Naour, G., Capron, F.: Automated mitosis detection using texture, sift features and hmax biologically inspired approach. Journal of Pathology Informatics **4**(Suppl) (2013) 10.4103/2153-3539.109870 PMC3678748 23766934

[123] Paul, A., Dey, A., Mukherjee, D.P., Sivaswamy, J., Tourani, V.: Regenerative random forest with automatic feature selection to detect mitosis in histopathological breast cancer images. In: International Conference on Medical Image Computing and Computer-Assisted Intervention. pp. 94–102. Springer (2015)

[124] Nateghi, R., Danyali, H., Helfroush, M.S.: Maximized inter-class weighted mean for fast and accurate mitosis cells detection in breast cancer histopathology images. Journal of Medical Systems **41**(9), 1–15 (2017) 10.1007/s10916-017-0773-9 28808813

[125] Al Rahhal, M.M.: Diagnoses of breast cancer in histopathlogical images based on deep learning. Journal of Theoretical and Applied Information Technology **97**(2) (2019)

[126] Aloyayri, A., Krzyżak, A.: Breast cancer classification from histopathological images using transfer learning and deep neural networks. In: International Conference on Artificial Intelligence and Soft Computing. pp. 491–502. Springer (2020)

[127] Anupama, M., Sowmya, V., Soman, K.: Breast cancer classification using capsule network with preprocessed histology images. In: 2019 International Conference on Communication and Signal Processing (ICCSP). pp. 0143–0147. IEEE (2019)

[128] Gaber, H., Mohamed, H., Ibrahim, M.: Breast cancer classification from histopathological images with separable convolutional neural network and parametric rectified linear unit. In: International Conference on Advanced Intelligent Systems and Informatics. pp. 370–382. Springer (2020)

[129] Gupta K, Chawla N (2020). Analysis of histopathological images for prediction of breast cancer using traditional classifiers with Pre-trained CNN. Procedia Computer Science.

[130] Saini M, Susan S (2020). Deep transfer with minority data augmentation for imbalanced breast cancer dataset. Applied Soft Computing.

[131] Jimenez-del Toro, O., Otálora, S., Andersson, M., Eurén, K., Hedlund, M., Rousson, M., Müller, H., Atzori, M.: Analysis of histopathology images: from traditional machine learning to deep learning. In: Biomedical Texture Analysis, pp. 281–314. Elsevier (2017)

[132] Gecer B, Aksoy S, Mercan E, Shapiro LG, Weaver DL, Elmore JG (2018). Detection and classification of cancer in whole slide breast histopathology images using deep convolutional networks. Pattern Recognition.

[133] Burçak, K.C., Baykan, Ö.K., Uğuz, H.: A new deep convolutional neural network model for classifying breast cancer histopathological images and the hyperparameter optimisation of the proposed model. The Journal of Supercomputing pp. 1–17 (2020)

[134] Han Z, Wei B, Zheng Y, Yin Y, Li K, Li S (2017). Breast cancer multi-classification from histopathological images with structured deep learning model. Scientific Reports.

[135] Zheng Y, Jiang Z, Xie F, Zhang H, Ma Y, Shi H, Zhao Y (2017). Feature extraction from histopathological images based on nucleus-guided convolutional neural network for breast lesion classification. Pattern Recognition.

[136] Cruz-Roa A, Gilmore H, Basavanhally A, Feldman M, Ganesan S, Shih NN, Tomaszewski J, González FA, Madabhushi A (2017). Accurate and reproducible invasive breast cancer detection in whole-slide images: A deep learning approach for quantifying tumor extent. Scientific Reports.

[137] Toğaçar M, Özkurt KB, Ergen B, Cömert Z (2020). Breastnet: A novel convolutional neural network model through histopathological images for the diagnosis of breast cancer. Physica A: Statistical Mechanics and its Applications.

[138] Li L, Pan X, Yang H, Liu Z, He Y, Li Z, Fan Y, Cao Z, Zhang L (2020). Multi-task deep learning for fine-grained classification and grading in breast cancer histopathological images. Multimedia Tools and Applications.

[139] Gour M, Jain S, Sunil Kumar T (2020). Residual learning based cnn for breast cancer histopathological image classification. International Journal of Imaging Systems and Technology.

[140] Yan R, Ren F, Wang Z, Wang L, Zhang T, Liu Y, Rao X, Zheng C, Zhang F (2020). Breast cancer histopathological image classification using a hybrid deep neural network. Methods.

[141] Jiang Y, Chen L, Zhang H, Xiao X (2019). Breast cancer histopathological image classification using convolutional neural networks with small se-resnet module. Plos One.

[142] Khan S, Islam N, Jan Z, Din IU, Rodrigues JJC (2019). A novel deep learning based framework for the detection and classification of breast cancer using transfer learning. Pattern Recognition Letters.

[143] Li X, Shen X, Zhou Y, Wang X, Li TQ (2020). Classification of breast cancer histopathological images using interleaved Densenet with SENet (IDSNet). Plos One.

[144] Du Y, Zhang R, Zargari A, Thai TC, Gunderson CC, Moxley KM, Liu H, Zheng B, Qiu Y (2018). Classification of tumor epithelium and stroma by exploiting image features learned by deep convolutional neural networks. Annals of Biomedical Engineering.

[145] Wang P, Song Q, Li Y, Lv S, Wang J, Li L, Zhang H (2020). Cross-task extreme learning machine for breast cancer image classification with deep convolutional features. Biomedical Signal Processing and Control.

[146] Bejnordi BE, Zuidhof G, Balkenhol M, Hermsen M, Bult P, van Ginneken B, Karssemeijer N, Litjens G, van der Laak J (2017). Context-aware stacked convolutional neural networks for classification of breast carcinomas in whole-slide histopathology images. Journal of Medical Imaging.

[147] Xie J, Liu R, Luttrell J, Zhang C (2019). Deep learning based analysis of histopathological images of breast cancer. Frontiers in Genetics.

[148] Celik Y, Talo M, Yildirim O, Karabatak M, Acharya UR (2020). Automated invasive ductal carcinoma detection based using deep transfer learning with whole-slide images. Pattern Recognition Letters.

[149] Sharma S, Mehra R (2020). Effect of layer-wise fine-tuning in magnification-dependent classification of breast cancer histopathological image. The Visual Computer.

[150] Alzubaidi L, Al-Shamma O, Fadhel MA, Farhan L, Zhang J, Duan Y (2020). Optimizing the performance of breast cancer classification by employing the same domain transfer learning from hybrid deep convolutional neural network model. Electronics.

[151] Xu B, Liu J, Hou X, Liu B, Garibaldi J, Ellis IO, Green A, Shen L, Qiu G (2019). Attention by selection: A deep selective attention approach to breast cancer classification. IEEE Transactions on Medical Imaging.

[152] Mewada HK, Patel AV, Hassaballah M, Alkinani MH, Mahant K (2020). Spectral-spatial features integrated convolution neural network for breast cancer classification. Sensors.

[153] Yang Z, Ran L, Zhang S, Xia Y, Zhang Y (2019). EMS-Net: Ensemble of multiscale convolutional neural networks for classification of breast cancer histology images. Neurocomputing.

[154] Kausar T, Wang M, Idrees M, Lu Y (2019). HWDCNN: Multi-class recognition in breast histopathology with haar wavelet decomposed image based convolution neural network. Biocybernetics and Biomedical Engineering.

[155] Yang H, Kim JY, Kim H, Adhikari SP (2019). Guided soft attention network for classification of breast cancer histopathology images. IEEE Transactions on Medical Imaging.

[156] Roy K, Banik D, Bhattacharjee D, Nasipuri M (2019). Patch-based system for classification of breast histology images using deep learning. Computerized Medical Imaging and Graphics.

[157] Nazeri, K., Aminpour, A., Ebrahimi, M.: Two-stage convolutional neural network for breast cancer histology image classification. In: International Conference Image Analysis and Recognition. pp. 717–726. Springer (2018)

[158] Bejnordi BE, Mullooly M, Pfeiffer RM, Fan S, Vacek PM, Weaver DL, Herschorn S, Brinton LA, van Ginneken B, Karssemeijer N (2018). Using deep convolutional neural networks to identify and classify tumor-associated stroma in diagnostic breast biopsies. Modern Pathology.

[159] Boumaraf S, Liu X, Zheng Z, Ma X, Ferkous C (2021). A new transfer learning based approach to magnification dependent and independent classification of breast cancer in histopathological images. Biomedical Signal Processing and Control.

[160] Jafarbiglo, S.K., Danyali, H., Helfroush, M.S.: Nuclear atypia grading in histopathological images of breast cancer using convolutional neural networks. In: 2018 4th Iranian Conference on Signal Processing and Intelligent Systems (ICSPIS). pp. 89–93. IEEE (2018)

[161] Kausar, T., Wang, M., Malik, M.: Cancer detection in breast histopathology with convolution neural network based approach. In: 2019 IEEE/ACS 16th International Conference on Computer Systems and Applications (AICCSA). pp. 1–5. IEEE (2019)

[162] Tripathi S, Singh SK, Lee HK (2021). An end-to-end breast tumour classification model using context-based patch modelling-a BiLSTM approach for image classification. Computerized Medical Imaging and Graphics.

[163] Albarqouni S, Baur C, Achilles F, Belagiannis V, Demirci S, Navab N (2016). Aggnet: Deep learning from crowds for mitosis detection in breast cancer histology images. IEEE Transactions on Medical Imaging.

[164] Balkenhol MC, Tellez D, Vreuls W, Clahsen PC, Pinckaers H, Ciompi F, Bult P, van der Laak JA (2019). Deep learning assisted mitotic counting for breast cancer. Laboratory Investigation.

[165] Jiménez, G., Racoceanu, D.: Deep learning for semantic segmentation vs. classification in computational pathology: Application to mitosis analysis in breast cancer grading. Frontiers in Bioengineering and Biotechnology **7**, 145 (2019) 10.3389/fbioe.2019.00145 PMC6597878 31281813

[166] Veta M, Van Diest PJ, Willems SM, Wang H, Madabhushi A, Cruz-Roa A, Gonzalez F, Larsen AB, Vestergaard JS, Dahl AB (2015). Assessment of algorithms for mitosis detection in breast cancer histopathology images. Medical Image Analysis.

[167] Mahmood T, Arsalan M, Owais M, Lee MB, Park KR (2020). Artificial intelligence-based mitosis detection in breast cancer histopathology images using faster R-CNN and Deep CNNs. Journal of Clinical Medicine.

[168] Wu, B., Kausar, T., Xiao, Q., Wang, M., Wang, W., Fan, B., Sun, D.: FF-CNN: An efficient deep neural network for mitosis detection in breast cancer histological images. In: Annual Conference on Medical Image Understanding and Analysis. pp. 249–260. Springer (2017)

[169] Wahab N, Khan A, Lee YS (2017). Two-phase deep convolutional neural network for reducing class skewness in histopathological images based breast cancer detection. Computers in Biology and Medicine.

[170] Wan T, Cao J, Chen J, Qin Z (2017). Automated grading of breast cancer histopathology using cascaded ensemble with combination of multi-level image features. Neurocomputing.

[171] Mehra R (2018). Breast cancer histology images classification: Training from scratch or transfer learning?. ICT Express.

[172] Bardou D, Zhang K, Ahmad SM (2018). Classification of breast cancer based on histology images using convolutional neural networks. IEEE Access.

[173] Nahid, A.A., Mehrabi, M.A., Kong, Y.: Histopathological breast cancer image classification by deep neural network techniques guided by local clustering. BioMed Research International **2018** (2018) 10.1155/2018/2362108 PMC5863327 29707566

[174] George K, Faziludeen S, Sankaran P (2020). Breast cancer detection from biopsy images using nucleus guided transfer learning and belief based fusion. Computers in Biology and Medicine.

[175] Pei, Z., Cao, S., Lu, L., Chen, W.: Direct cellularity estimation on breast cancer histopathology images using transfer learning. Computational and Mathematical Methods in Medicine **2019** (2019) 10.1155/2019/3041250 PMC6590493 31281408

[176] Radiya-Dixit E, Zhu D, Beck AH (2017). Automated classification of benign and malignant proliferative breast lesions. Scientific Reports.

[177] Wang Y, Lei B, Elazab A, Tan EL, Wang W, Huang F, Gong X, Wang T (2020). Breast cancer image classification via multi-network features and dual-network orthogonal low-rank learning. IEEE Access.

[178] Vo DM, Nguyen NQ, Lee SW (2019). Classification of breast cancer histology images using incremental boosting convolution networks. Information Sciences.

[179] Sharma, S., Mehra, R.: Conventional machine learning and deep learning approach for multi-classification of breast cancer histopathology images–a comparative insight. Journal of Digital Imaging **33**(3), 632–654 (2020) 10.1007/s10278-019-00307-y PMC7256154 31900812

[180] Saxena S, Shukla S, Gyanchandani M (2020). Pre-trained convolutional neural networks as feature extractors for diagnosis of breast cancer using histopathology. International Journal of Imaging Systems and Technology.

[181] Wang H, Roa AC, Basavanhally AN, Gilmore HL, Shih N, Feldman M, Tomaszewski J, Gonzalez F, Madabhushi A (2014). Mitosis detection in breast cancer pathology images by combining handcrafted and convolutional neural network features. Journal of Medical Imaging.

[182] Saha M, Chakraborty C, Racoceanu D (2018). Efficient deep learning model for mitosis detection using breast histopathology images. Computerized Medical Imaging and Graphics.

[183] Beevi KS, Nair MS, Bindu G (2019). Automatic mitosis detection in breast histopathology images using convolutional neural network based deep transfer learning. Biocybernetics and Biomedical Engineering.

[184] Dodballapur, V., Song, Y., Huang, H., Chen, M., Chrzanowski, W., Cai, W.: Mask-driven mitosis detection in histopathology images. In: 2019 IEEE 16th International Symposium on Biomedical Imaging (ISBI 2019). pp. 1855–1859. IEEE (2019)

[185] Zhang, Y., Zhang, B., Lu, W.: Breast cancer classification from histological images with multiple features and random subspace classifier ensemble. In: AIP Conference Proceedings. vol. 1371, pp. 19–28. American Institute of Physics (2011)

[186] Cohen, S.: Artificial Intelligence and Deep Learning in Pathology E-Book. Elsevier Health Sciences (2020)

